# The Anthelmintic Activity of Praziquantel Analogs Correlates with Structure–Activity Relationships at TRPM_PZQ_ Orthologs

**DOI:** 10.1021/acsmedchemlett.3c00350

**Published:** 2023-10-25

**Authors:** Daniel J. Sprague, Marc Kaethner, Sang-Kyu Park, Claudia M. Rohr, Jade L. Harris, David Maillard, Thomas Spangenberg, Britta Lundström-Stadelmann, Jonathan S. Marchant

**Affiliations:** † Department of Cell Biology, Neurobiology, and Anatomy, Medical College of Wisconsin, Milwaukee, Wisconsin 53226, United States; ‡ Program in Chemical Biology, Department of Biochemistry, Medical College of Wisconsin, Milwaukee, Wisconsin 53226, United States; § Institute of Parasitology, Department of Infectious Diseases and Pathobiology, Vetsuisse Faculty, University of Bern, 3012 Berne, Switzerland; ∥ Graduate School for Cellular and Biomedical Sciences, University of Bern, 3012 Berne, Switzerland; ⊥ Central Process Development - Downstream Processing Services, Merck Electronics KGaA, Frankfurter Strasse 250, 64293 Darmstadt, Germany; # Global Health Institute of Merck, Ares Trading S.A., a subsidiary of Merck KGaA, Darmstadt, Germany, 1262 Eysins, Switzerland; ∇ Multidisciplinary Center for Infectious Diseases, University of Bern, 3012 Berne, Switzerland

**Keywords:** Parasitic flatworm, Schistosome, Tapeworm, TRP channel, Ion channel

## Abstract

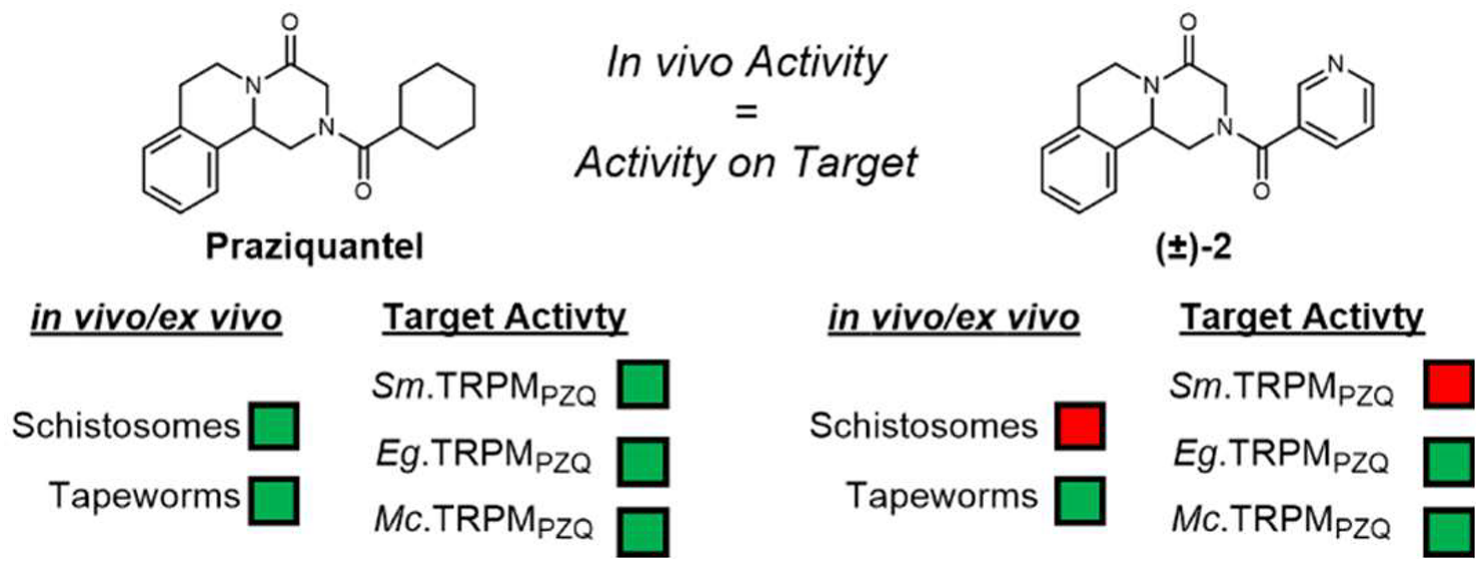

The anthelmintic drug praziquantel remains a key clinical therapy for treating various diseases caused by parasitic flatworms. The parasite target of praziquantel has remained undefined despite longstanding usage in the clinic, although a candidate ion channel target, named TRPM_PZQ_, has recently been identified. Intriguingly, certain praziquantel derivatives show different activities against different parasites: for example, some praziquantel analogs are considerably more active against cestodes than against schistosomes. Here we interrogate whether the different activities of praziquantel analogs against different parasites are also reflected by unique structure–activity relationships at the TRPM_PZQ_ channels found in these different organisms. To do this, several praziquantel analogs were synthesized and functionally profiled against schistosome and cestode TRPM_PZQ_ channels. Data demonstrate that structure–activity relationships are closely mirrored between parasites and their TRPM_PZQ_ orthologs, providing further support for TRPM_PZQ_ as the therapeutically relevant target of praziquantel.

Exactly 40 years ago, a highly influential review on the anthelmintic activity of praziquantel (PZQ) was published by Peter Andrews and Herbert Thomas (both at Bayer AG) and Rolf Pohlke and Jürgen Seubert (both at E. Merck KG).^1^ That work detailed the discovery of the anthelmintic activity of PZQ, derivatization of the scaffold, the drug’s pharmacokinetic and safety profile, and the broad efficacy of this new therapeutic agent against a range of parasitic flatworms. The summary of data interrogating the activity of different pyrazino[2,1-*a*]isoquinoline derivatives against a representative trematode (*Schistosoma mansoni*) and cestode model (*Hymenolepis nana*) established both the “tightness” of the pharmacophore that underpins the efficacy of PZQ and a “structure–activity” fingerprint for the action of this drug that has long served as a reference standard for the field.

Following decades of clinical usage of PZQ for treatment of various diseases caused by parasitic flatworms,^2−5^ a candidate target was recently identified^6^ in *Schistosoma mansoni*. This target is a transient receptor potential (TRP) ion channel of the melastatin subfamily, named *Sm*.TRPM_PZQ_,^6−8^ that mirrors the structure–activity relationship (SAR) of PZQ derivatives^7^ as described by Andrews et al.^1^ TRPM_PZQ_ is a large nonselective cation channel unique to flatworms.^6,8−10^ Activation of *Sm*.TRPM_PZQ_ is thought to elicit excitotoxicity through membrane depolarization, spastic contraction, and surface damage to the parasite, which then catalyzes immunological clearance from infected hosts.^11^ Additional evidence from genetic association studies,^12^ pharmacological screening,^13^ and functional profiling of TRPM_PZQ_ orthologs^9,10^ add support for TRPM_PZQ_ serving as the therapeutically relevant parasite target of PZQ.

The work of Andrews et al., however, provides an additional opportunity to interrogate the candidacy of TRPM_PZQ_.^1^ From the extensive list of PZQ derivatives described by Andrews et al.,^1^ a small number of analogs displayed good activity against cestodes but not against schistosomes. These analogs of PZQ encompassed replacement of the cyclohexyl group by 3-pyridyl ((±)-**2**, (*R*)-**2**, and (*S*)-**2**), 4-nitrophenyl (**3**), 4-*N*-methylaniline (**4**), 4-*N*,*N*-dimethylaniline (**5**), 4-aniline (**6**), *cis*-4-aminocyclohexyl (**7**) and -cyclopropyl (**8**) groups (Chart 1). Do such analogs, which exhibit differential antiparasitic activity *versus* cestodes and schistosomes, display differential activity at schistosome and cestode TRPM_PZQ_? If the specific SAR of PZQ analogs against these different parasites mimics the SAR at the corresponding TRPM_PZQ_ channel, these data would provide further support for TRPM_PZQ_ as the clinically relevant *in vivo* target of PZQ. To tackle this question, we synthesized eight PZQ analogs from Andrews et al.^1^ that exhibited divergent bioactivity against schistosomes and cestodes and profiled them against schistosome TRPM_PZQ_ and two cestode TRPM_PZQ_ representatives.^10^

**Chart 1:**
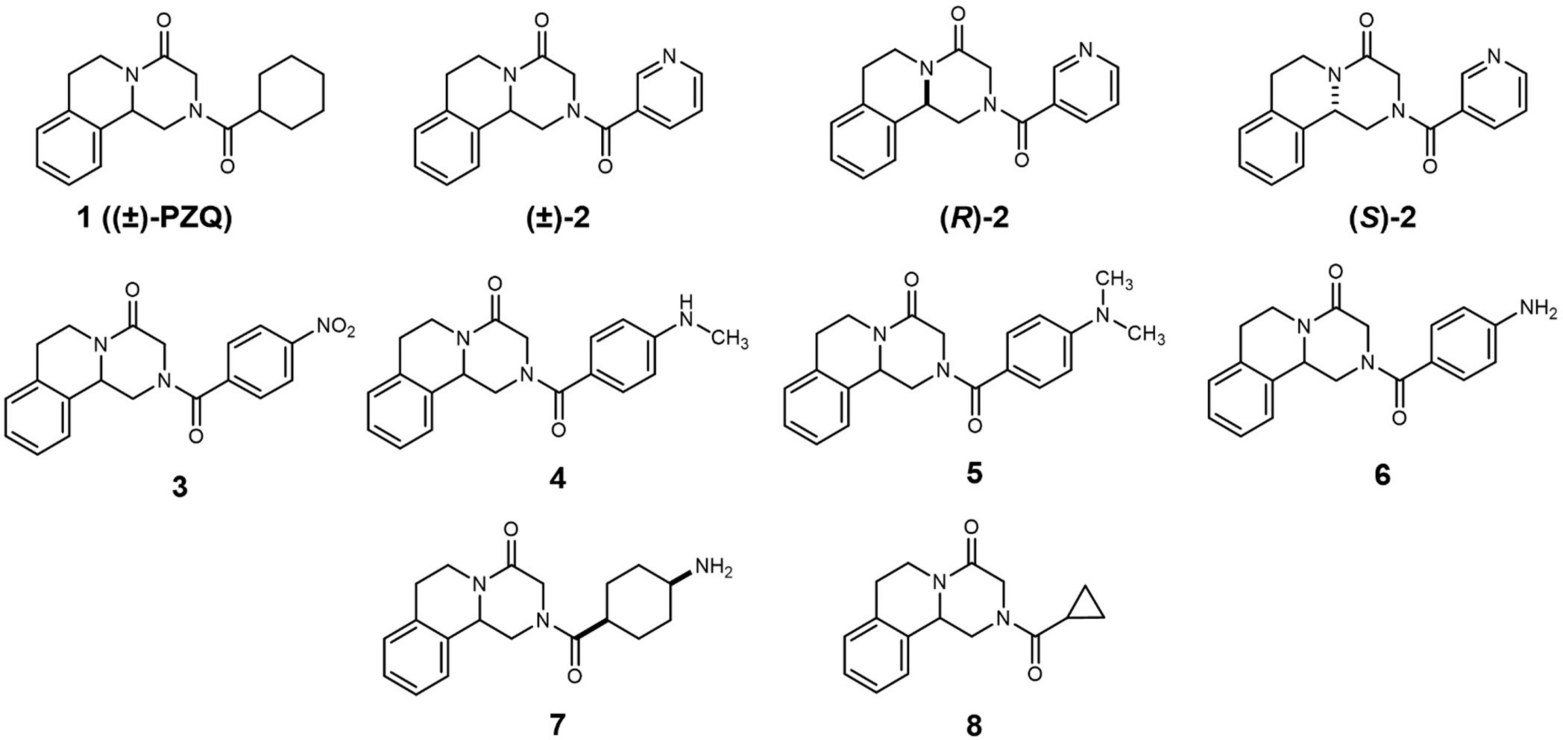
Structures of the PZQ Analogs Studied in This Work

Table 1 (columns 2–4) reproduces data from Andrews et al.^1^ that scored the activity of PZQ derivatives against *Schistosoma mansoni* and *Hymenolepis nana*. Data were collected against *S. mansoniin vitro* and *in vivo* using a mouse model and also against *H. nana* using an *in vivo* mouse model.^1^ Results were previously scored using broad potency ranges, graded “+++”, “++”, “+”, or “0” (see Table 1 legend). While there are caveats in the interpretation of these data, it is evident that the selected analogs displayed appreciable activity in the cestode model (column 4) but lower or minimal activity when tested against schistosomes (columns 2 and 3). In contrast, (±)-PZQ (**1**) showed equivalent activity in phenotypic grading across all of the bioassays, with preferential stereoselectivity toward the *R* enantiomer (Table 1).

**Table 1:** Comparison of Antischistosomal and Anticestodal Activities of PZQ Derivatives with Their Activities at the Corresponding TRPM_PZQ_ Orthologs^a^

	previous studies					
	*S. mansoni*		*H. nana*	this work: EC_50_ (μM)		
compound	*in vitro*	*in vivo*	*in vivo*	*Sm*.TRPM_PZQ_	*Eg*.TRPM_PZQ_	*Mc*.TRPM_PZQ_
**1** ((±)-PZQ)	+++	+++	+++	0.65 ± 0.057	0.10 ± 0.009	0.12 ± 0.016
((*R*)-PZQ)^b^	+++	+++	+++	0.28 ± 0.03	0.05 ± 0.01	0.08 ± 0.003
((*S*)-PZQ)^b^	++	+	++	28 ± 2.8	0.78 ± 0.13	1.23 ± 0.17
(±)-**2**	+	+	+++	inactive	2.3 ± 1.1	5.3 ± 1.3
(*R*)-**2**	0	++	+++	>100	1.4 ± 0.11	0.98 ± 0.088
(*S*)-**2**	0	0	++	inactive	22 ± 2.3	16 ± 2.2
**3**	0	+	++	inactive	inactive	inactive
**4**	0	++	+++	inactive	>100	>100
**5**	0	++	+++	inactive	inactive	inactive
**6**	++	++	+++	12 ± 2.6	0.96 ± 0.14	0.80 ± 0.22
**7**	0	0	+	inactive	>100	>100
**8**	0	+	++	inactive	53 ± 16	65 ± 14

a All presented *in vitro* and *in vivo* data on organisms (columns 2–4) were taken from ref (1). For *in vitro* studies using *S. mansoni*, “+++” indicates a maximal effect ≤3.2 μM, “++” indicates a maximal effect ≤320 μM, “+” indicates a less than maximal effect at ≤320 μM, and “0” indicates no effect at ≤320 μM. For *in vivo* studies using *S. mansoni*, “+++” indicates a complete reduction of worms at 50 mg/kg dosing (×5), “++” indicates complete reduction of worms at 500 mg/kg dosing (×5), “+” indicates less than 90% worm reduction at 500 mg/kg dosing (×5), and “0” indicates no effect at 500 mg/kg dosing (×5). For *in vivo* studies using *H. nana*, “+++” indicates a complete reduction of worms at 25 mg/kg dosing (×1), “++” indicates complete reduction of worms at 500 mg/kg dosing (×1), “+” indicates less than 90% worm reduction at 500 mg/kg dosing (×1), and “0” indicates no effect at 500 mg/kg dosing (×1). Columns 5–7 tabulate the EC_50_ values for analog activation of trematode and cestode TRPM_PZQ_ orthologs *in vitro*. Data are shown as mean ± SEM for *n* ≥ 3 independent transfections. *Eg*.TRPM_PZQ_ = *Echinococcus granulosus* TRPM_PZQ_; *Mc*.TRPM_PZQ_ = *Mesocestoides corti* TRPM_PZQ_.

b EC_50_ values on the channel are reported in ref (10) and are provided for completeness.

Based on Andrews’ data,^1^ we resynthesized compounds **2**–**8**. These analogs were then tested for activity at *Sm*.TRPM_PZQ_ and two representative cestode TRPM_PZQ_ orthologs (*Echinococcus granulosus* TRPM_PZQ_ (*Eg*.TRPM_PZQ_) and *Mesocestoides corti* TRPM_PZQ_ (*Mc*.TRPM_PZQ_)) that have been successfully heterologously expressed.^10^ This was done using a fluorescence-based reporter assay to measure changes in cytosolic Ca^2+^ in HEK293 cells transiently expressing the individual TRPM_PZQ_ ion channels. Results from these assays are presented in Figure 1, and all EC_50_ values are tabulated in Table 1 (columns 5–7).

**Figure 1:**
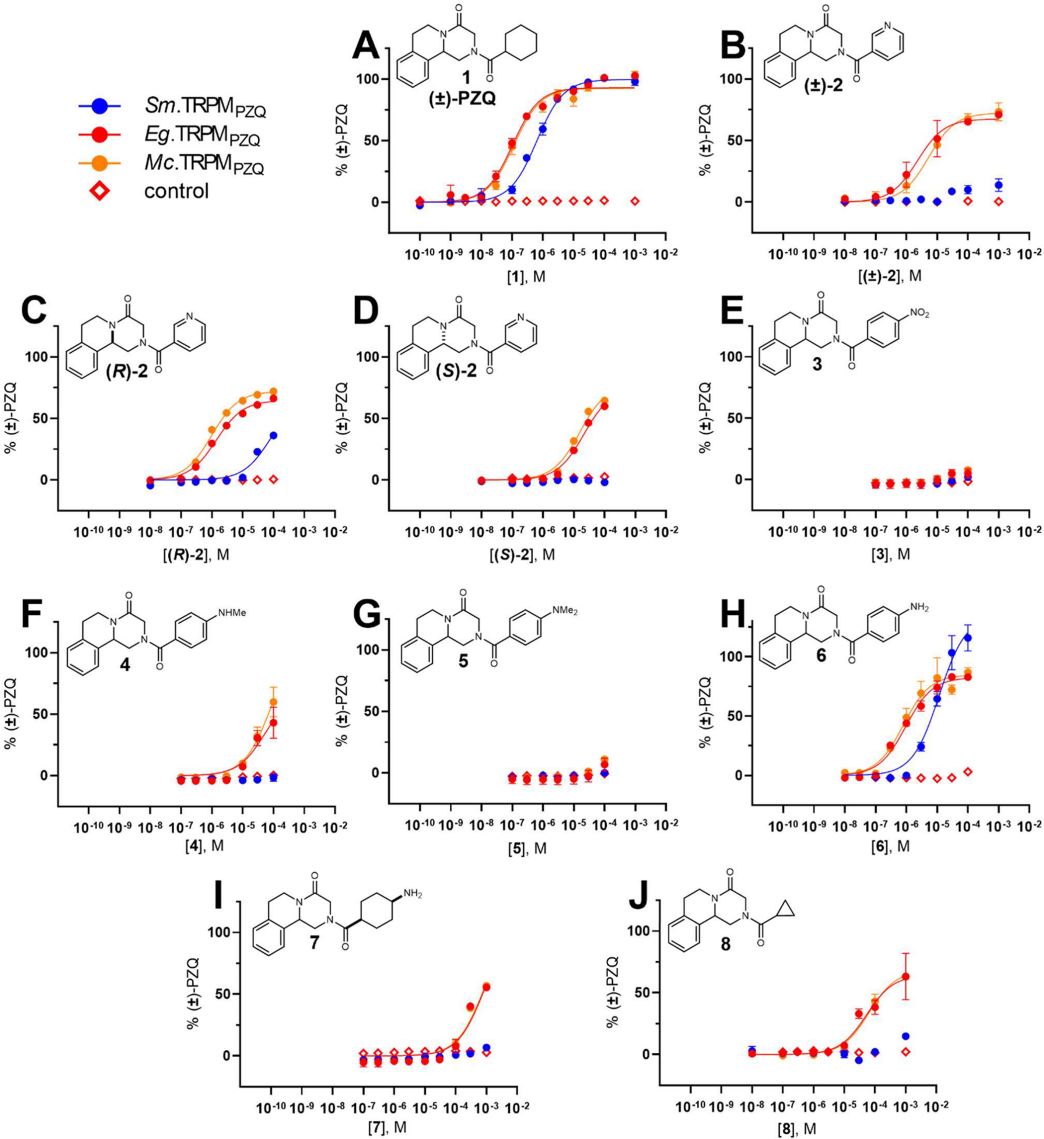
Functional profiling of PZQ analogs against different TRPM_PZQ_ channels. Shown are concentration–response relationships for *Sm*.TRPM_PZQ_ (blue circles), *Eg*.TRPM_PZQ_ (red circles), and *Mc*.TRPM_PZQ_ (orange circles) in response to increasing concentrations of PZQ analogs. Responses to molecules in untransfected HEK293 cells are shown as controls (red diamonds).

Data for (±)-PZQ (**1**) are shown in Figure 1A. All three TRPM_PZQ_ channels, *Sm*.TRPM_PZQ_ (EC_50_ = 645 ± 57 nM), *Eg*.TRPM_PZQ_ (EC_50_ = 104 ± 9 nM), and *Mc*.TRPM_PZQ_ (EC_50_= 112 ± 16 nM), were potently activated by **1**. The cestode TRPM_PZQ_ channels displayed ∼6-fold higher sensitivity to PZQ, consistent with previous reports and the known sensitivity of many cestode species to PZQ.^6,7,10^Figure 1B–D shows results for the 3-pyridyl PZQ analogs. The racemate, (±)-**2**, showed little activity at *Sm*.TRPM_PZQ_ but displayed low-micromolar potency at both cestode channels (EC_50_ for *Eg*.TRPM_PZQ_ = 2.3 ± 1.1 μM, EC_50_ for *Mc*.TRPM_PZQ_ = 5.3 ± 1.3 μM; Figure 1B). Consistent with the previously detailed activity of PZQ enantiomers *in vivo*(14) that is also mirrored at TRPM_PZQ_^6^ (Table 1, entries 2 and 3), the *R* enantiomer (*R*)-**2** was more potent than the *S* enantiomer (*S*)-**2** at the cestode channels (Figure 1C vs Figure 1D). All activity at *Sm*.TRPM_PZQ_ was attributed to enantiomer (*R*)-**2** (Figure 1C). Activation of both cestode channels, with negligible activity at the schistosome channel, was consistent with the Andrews et al. grading classification (Table 1).^1^

A series of substituted phenyl derivatives were then profiled. In previous phenotypic assays, these analogs were active against *H. nana* and *S. mansoniin vivo* but were inactive against *S. mansoniin vitro* (Table 1).^1^ Consistent with these prior observations, analogs **3**–**5** showed no activity at *Sm*.TRPM_PZQ_ (Figure 1E–G), and the activity of these analogs at cyclophyllidean cestode TRPM_PZQ_ orthologs was also low. As previously proposed,^1^ the *in vivo* activity of compounds **3**–**5** is likely caused by dealkylative metabolism to aniline **6**, and subsequent synthesis and profiling of **6** confirmed this (Figure 1H). Compound **6** activated *Sm*.TRPM_PZQ_ (EC_50_ = 11.6 ± 2.6 μM), *Eg*.TRPM_PZQ_ (EC_50_ = 957 ± 141 nM), and *Mc*.TRPM_PZQ_ (EC_50_ = 796 ± 224 nM), and displayed an ∼8–10-fold increase in potency at cestode TRPM_PZQ_ compared to schistosome TRPM_PZQ_. This was similar to the ∼6-fold increase in potency for (±)-PZQ (**1**) at cestode *versus* schistosome TRPM_PZQ_.

Finally, we profiled modifications of the cyclohexyl group of PZQ. Compound **7**, a 4′-aminocyclohexyl derivative, lacked activity at *Sm*.TRPM_PZQ_ and was only weakly active at cestode TRPM_PZQ_ orthologs (Figure 1I), corresponding to the weak *in vivo* activity against *H. nana* previously reported (Table 1).^1^ Finally, cyclopropyl analog **8** activated both cestode TRPM_PZQ_ representatives at concentrations >10 μM (EC_50_ = 53 ± 16 μM for *Eg*.TRPM_PZQ_, EC_50_ = 65 ± 14 μM for *Mc*.TRPM_PZQ_; Figure 1J). Little activity was observed at *Sm*.TRPM_PZQ_. These target-based data are again consistent with the phenotypic observations of Andrews et al. (Table 1).^1^

Overall, from the profiled PZQ analogs, only a single analog (compound **6**) was sufficiently active at *Sm*.TRPM_PZQ_ to derive an EC_50_ value, while five analogs displayed activity at the cestode channels (Table 1). Analog **6** was not one of the eight PZQ analogs selected based on the differential potency between cestodes and schistosomes but was synthesized to explain the *in vivo* activity of the other analogs. Therefore, the different potencies of these analogs against cestodes and schistosomes, seen in phenotypic the data of Andrews et al.^1^ 40 years ago, was mirrored by the same differential potency of these analogs in target-based assays at the different TRPM_PZQ_ channels. Some caveats are however appropriate.

First, the original data did not report the activity of the PZQ derivatives against cestodes *ex vivo* (*in vitro*), so in selecting these analogs, there was no direct comparator for the action of all of these analogs between schistosomes and cestodes. Therefore, two analogs (*R*)-**2** and (*S*)-**2** were tested on *Echinococcus multilocularis* protoscoleces for comparison with PZQ (Figure 2A–D). When compared with the vehicle control, treatment with (±)-PZQ (1 μM) caused a sustained contraction of the protoscoleces (Figure 2A,B). When protoscoleces were treated with the 3-pyridyl enantiomers (*R*)-**2** (Figure 2C) and (*S*)-**2** (Figure 2D), a similar contraction was observed, with (*R*)-**2** being more potent than (*S*)-**2**. To determine IC_50_ values, concentration–response curves were obtained at multiple time points (Figure 2E–G). Low concentrations of PZQ and the 3-pyridyl analogs stimulated motility at the early time points (Figure 2E). After 24 h, the IC_50_ for (*R*)-**2** was ∼3 μM, and the IC_50_ for (*S*)-**2** was ∼30 μM (Figure 2G). Prolonged treatment with (±)-PZQ proved toxic after 24 h, and therefore, a more realistic IC_50_ value (∼100 nM) was calculated at 12 h postincubation (Figure 2F). This mirrors the EC_50_ at a cestode TRPM_PZQ_ of 100 nM (Table 1). These data for activity against cestodes *ex vivo* are again consistent with the potencies of the molecules at cestode TRPM_PZQ_ (Table 1).

**Figure 2:**
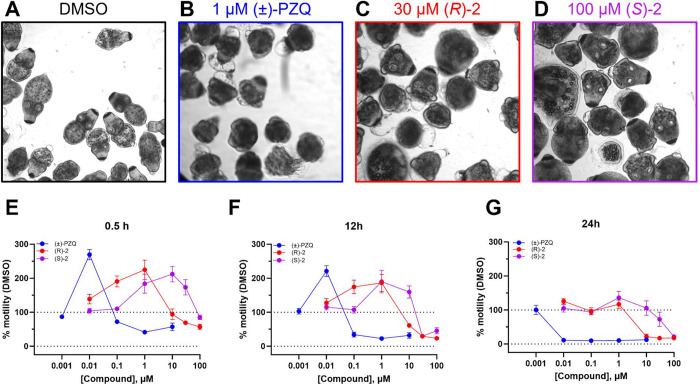
*E. multilocularis* protoscoleces after treatment with (A) DMSO, (B) (±)-PZQ (1 μM), (C) (*R*)-**2** (30 μM), and (D) (*S*)-**2** (100 μM). (E**–**G) Concentration–response motility graphs of *E. multilocularis* protosoleces after treatment with (±)-PZQ (blue circles), (*R*)-**2** (red circles), and (*S*)-**2** (purple circles) after (E) 0.5 h, (F) 12 h, and (G) 24 h. Data are plotted as % motility *vs* vehicle (DMSO) control (mean ± SEM).

Second, the cestode TRPM_PZQ_ and motility assays derive from different cyclophyllidean cestodes (*E. granulosus*, *M. corti*, and *E. multilocularis*) than the model (*H. nana*) used by Andrews et al.^1^ However, we note that the amino acid residues lining the PZQ binding pocket of TRPM_PZQ_ are identical across all cyclophyllidean cestode TRPM_PZQ_ orthologs examined to date, including *H. nana* TRPM_PZQ_ (Figure S1).^10^ This is consistent with the similarity of the functional data from *Eg*.TRPM_PZQ_ and *Mc*.TRPM_PZQ_.

Considering the structures of the analogs profiled here, it is evident that the cestode TRPM_PZQ_ binding pocket is more tolerant to substitutions of the cyclohexyl moiety of PZQ than is the schistosome TRPM_PZQ_ binding pocket. Aniline analog **6** and pyridyl analog (*R*)-**2** show submicromolar potency, and even the cyclopropyl analog **8**, which displayed no activity at *Sm*.TRPM_PZQ_ at 100 μM, was clearly active at the cestode TRPM_PZQ_ orthologs, consistent with the differential activity seen by Andrews et al. (Table 1).^1^

This increased tolerability to modifications of the cyclohexyl group of PZQ, a key part of the pharmacophore at *Sm*.TRPM_PZQ_,^7^ may provide opportunity to accommodate other cyclohexane ring modifications—notably, more metabolically stable PZQ derivatives—within the cestode TRPM_PZQ_ binding pocket.^15,16^ This could potentially enhance the *in vivo* efficacy of these analogs for treating cestode infections, and these data therefore highlight an opportunity to design drugs that selectively target cestode TRPM_PZQ_. Whether the absolute potency of such analogs can be further improved over PZQ to yield better treatments for cestode species less sensitive to PZQ (*e.g.*, noncyclophyllidean cestodes^10,17^) or for cestode life cycle stages that are hard to treat will require further work and a better understanding of the molecular basis by which cestode-selective analogs engage the TRPM_PZQ_ binding pocket. Such understanding will be aided by the recent mapping of the PZQ binding pocket in TRPM_PZQ_ orthologs in different parasitic flatworms and a capacity to model these interactions.^7,10^ Of likely relevance are two natural amino acid variants—a histidine residue in the S1 transmembrane helix and a serine residue in the S4/S5 linker—that are different between the PZQ binding pocket of trematode and cyclophyllidean cestode TRPM_PZQ_ (compare Figure 3A and Figure 3B).^10^ This natural variation within the TRPM_PZQ_ binding pocket provides a possible molecular explanation underpinning the differential SAR of the PZQ analogs. Natural variation in the binding pocket has previously been shown to render *Fasciola* spp. TRPM_PZQ_ insensitive to PZQ.^7,10^

**Figure 3:**
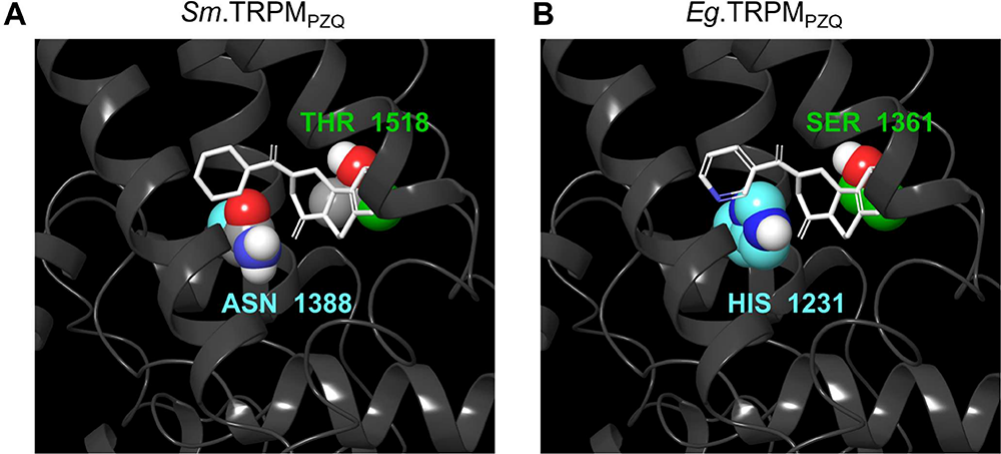
(A) Predicted binding pose of (*R*)-PZQ (white) in *Sm*.TRPM_PZQ_. (B) Visualization of (*R*)-**2** (white) adjacent to residues showing variation in *Eg*.TRPM_PZQ_, based on (A). Residues showing variation between the channels are highlighted in TM1 (Asn 1388/His 1231, teal) and TM4 (T1518/S1361, green).

The activity of (*R*)-**2** at cestode TRPM_PZQ_*versusSm*.TRPM_PZQ_ is noteworthy in the context of this natural variation. The transmembrane helix 1 (S1) variation occurs at a position in close proximity to the pyridyl nitrogen (Figure 3B), and it is conceivable that there is an electrostatic interaction between the histidine residue in cestode TRPM_PZQ_ (*e.g.*, H1231 in *Eg*.TRPM_PZQ_) and the pyridine ring that is absent with the uncharged asparagine residue in schistosome TRPM_PZQ_ (*e.g.*, N1388 in *Sm*.TRPM_PZQ_). Interactions between PZQ and this S1 residue are important for PZQ activation of TRPM_PZQ_ across species.^7,10^

In summary, functional profiling of various PZQ derivatives on parasitic flatworms and at their respective TRPM_PZQ_ orthologs shows that “the glove fits”: the SAR between different parasites and different parasite TRPM_PZQ_ orthologs matches well. These data provide additional support for TRPM_PZQ_ serving as the relevant therapeutic target of PZQ in parasitic flatworms.

## Supporting Information Available

Sequence alignment of the PZQ binding pocket residues in cyclophyllidean cestodes; materials and methods, synthetic procedures, characterization data, and ^1^H/^13^C NMR spectra for all previously uncharacterized molecules (PDF)

## Abbreviations

PZQ: praziquantel
TRP: transient receptor potential
*Sm*.TRPM_PZQ_: *Schistosoma mansoni* transient receptor potential praziquantel
*S. mansoni*: *Schistosoma mansoni*
*H. nana*: *Hymenolepis nana*
*Eg*.TRPM_PZQ_: *Echinococcus granulosus* transient receptor potential praziquantel
*Mc*.TRPM_PZQ_: *Mesocestoides corti* transient receptor potential praziquantel
*E. multilocularis*: *Echinococcus multilocularis*
*E. granulosus*: *Echinococcus granulosus*
*M. corti*: *Mesocestoides corti*
DMEM: Dulbecco’s modified Eagle’s medium
FBS: fetal bovine serum
FLIPR: fluorescence imaging plate reader
HBSS: Hanks’ balanced salt solution
HEPES: 4-(2-hydroxyethyl)-1-piperazineethanesulfonic acid
