# Supplementary material for: The Anthelmintic Activity of Praziquantel Analogs Correlates with Structure–Activity Relationships at TRPMPZQ Orthologs
Source: ACS Med Chem Lett. 2023 Oct 25;14(11):1537–43. doi: 10.1021/acsmedchemlett.3c00350 (PMC10641913; doi:10.1021/acsmedchemlett.3c00350)
Supplement: Supplementary file 1 — ml3c00350_si_001.pdf [file ml3c00350_si_001.pdf]

The Anthelmintic Activity of Praziquantel Analogs Correlates with Structure-Activity Relationships at $TRPM_{PZQ}$ Orthologs

Daniel J. Sprague[1,2]*, Marc Kaethner[3,4], Sang-Kyu Park[1], Claudia M. Rohr[1], Jade L. Harris[1], David Maillard[5], Thomas Spangenberg[6], Britta Lundström-Stadelmann[3,7], and Jonathan S. Marchant[1]*

[1]Department of Cell Biology, Neurobiology, and Anatomy, Medical College of Wisconsin, Milwaukee, WI 53226, USA
[2]Program in Chemical Biology, Department of Biochemistry, Medical College of Wisconsin, Milwaukee, WI 53226, USA
[3]Institute of Parasitology, Department of Infectious Diseases and Pathobiology, Vetsuisse Faculty, University of Bern, 3012 Berne, Switzerland
[4]Graduate School for Cellular and Biomedical Sciences, University of Bern, 3012 Berne, Switzerland.
[5] Central Process Development - Downstream Processing Services, Merck Electronics KGaA, Frankfurter Strasse 250, 64293 Darmstadt, Germany.
[6]Global Health Institute of Merck, Ares Trading S.A., a subsidiary of Merck KGaA, Darmstadt, Germany, 1262 Eysins, Switzerland.
[7]Multidisciplinary Center for Infectious Diseases, University of Bern, 3012 Berne, Switzerland.

SI-X

Figure S1 3
Materials and Methods 4
General Remarks 4
Cell Culture and transfection 4
Ca^{2+} imaging assays 4
Cestode motility assays 4
Computational Modeling 4
Synthetic Chemistry Procedures 5
General Remarks 5
(±)-2-Nicotinoyl-1,2,3,6,7,11b-hexahydro-4*H*-pyrazino[2,1-*a*]isoquinolin-4-one ((±)-2) 5
(*R*)-2-Nicotinoyl-1,2,3,6,7,11b-hexahydro-4*H*-pyrazino[2,1-*a*]isoquinolin-4-one ((*R*)-2) 5
(*S*)-2-Nicotinoyl-1,2,3,6,7,11b-hexahydro-4*H*-pyrazino[2,1-*a*]isoquinolin-4-one ((*S*)-2) 6
2-(4-Nitrobenzoyl)-1,2,3,6,7,11b-hexahydro-4*H*-pyrazino[2,1-*a*]isoquinolin-4-one (3) 6
2-(4-(Methylamino)benzoyl)-1,2,3,6,7,11b-hexahydro-4*H*-pyrazino[2,1-*a*]isoquinolin-4-one (4) 6
2-(4-(Dimethylamino)benzoyl)-1,2,3,6,7,11b-hexahydro-4*H*-pyrazino[2,1-*a*]isoquinolin-4-one (5) 7
2-(4-Aminobenzoyl)-1,2,3,6,7,11b-hexahydro-4*H*-pyrazino[2,1-*a*]isoquinolin-4-one (6) 7
2-(4-Aminocyclohexane-1-carbonyl)-1,2,3,6,7,11b-hexahydro-4*H*-pyrazino[2,1-*a*]isoquinolin-4-one (7) 7
(*R*)-Praziquanamine (S3) 8
(*S*)-Praziquanamine (S4) 8
Figure S2. ^{1}H NMR (500 MHz, DMSO-d_6) of (±)-2 9
Figure S3. ^{13}C NMR (125 MHz, DMSO-d_6) of (±)-2 10
Figure S4. ^{1}H NMR (500 MHz, DMSO-d_6) of 3 11
Figure S5. ^{13}C NMR (125 MHz, DMSO-d_6) of 3 12
Figure S6. ^{1}H NMR (500 MHz, $CDCl_3$) of 4 13
Figure S7. ^{13}C NMR (125 MHz, $CDCl_3$) of 4 14
Figure S8. ^{1}H NMR (500 MHz, $CDCl_3$) of 5 15
Figure S9. ^{13}C NMR (125 MHz, $CDCl_3$) of 5 16
Figure S10. ^{1}H NMR (500 MHz, DMSO-d_6) of 6 17

Figure S11. ^{13}C NMR (125 MHz, DMSO-d_6) of 6 18

Figure S12. HRMS (ESI) of 6..... 19

Figure S13. ^{1}H NMR (500 MHz, DMSO-d_6) of 7 20

Figure S14. ^{13}C NMR (125 MHz, DMSO-d_6) of 7 21

Figure S15. HPLC trace of S3 22

Figure S16. HPLC trace of S4 23

```
Eg.TRPMPZQ   HTISYVLFLVLFSYLLLVDFKVRITGVEYIVLAWVITLFIEEIKQIAWSVLSGISFSTYI   60
Mc.TRPMPZQ   HTISYVLFLVIFSYLLLVDFKVKITGVEYIVLAWVITLFIEEIKQIAWSVLSGISFSTYI   60
Em.TRPMPZQ   HTISYVLFLVLFSYLLLVDFKVRITGVEYIVLAWVITLFIEEIKQIAWSVLSGISFSTYI   60
Tm.TRPMPZQ   HTISYVLFLVLFSYLLLVDFKVRITGVEYIVLAWVITLFIEEIKQIAWSVLSGISFSTYI   60
Ts.TRPMPZQ   HTISYVLFLVLFSYLLLVDFKVRITGVEYIVLAWVITLFIEEIKQIAWSVLSGISFSTYI   60
Hm.TRPMPZQ   HTISYVLFLVLFSYLLLVDFKVQMTGVEYIVLAWVITLFIEEVKQIAWSVLSGISFSTYI   60
Hn.TRPMPZQ   HTISYVLFLVLFSYLLLVDFKVKMTGVEYIVLAWVITLFIEEVKQIAWSVLSGISFSTYI   60
Me.TRPMPZQ   HTISYGLFLILFSYLLLVDFKVKMTGVEYIVLAWVITLFIEEIKQIAWSVLSGISFSTYI   60
Ht.TRPMPZQ   HTISYVLFLVLFSYLLLVDFKVRITGVEYIVLAWVITLFIEEIKQIAWSVLSGISFSTYI   60
             ***** ***::***********::******************:*****************

Eg.TRPMPZQ   SDGWNKLDCTGLILYIVGFILRLIVLARLGDSQLNHDTEAFHILTDPIMDPSRICMAFSL   120
Mc.TRPMPZQ   SDGWNKLDCTGLILYIIGFTLRLIVLARLGDSQMNNDTEAFHILTDPIMDPSRICMAFSL   120
Em.TRPMPZQ   SDGWNKLDCTGLILYIVGFILRLIVLARLGDSQLSHDTEAFHILTDPIMDPSRICMAFSL   120
Tm.TRPMPZQ   SDGWNKLDCTGLILYIVGFVLRLVVLARLGDSQLSHDTEAFHILTDPIMDPSRICMAFSL   120
Ts.TRPMPZQ   SDGWNKLDCTGLILYIVGFILRLVVLARLGDSQLSHDTEAFHILTDPIMDPSRICMAFSL   120
Hm.TRPMPZQ   SDGWNKLDCTGLILYIVGFILRLVVLARLGDSKMNSDTEAFHILTDPILDPSRICMAFAL   120
Hn.TRPMPZQ   SDGWNKLDCTGLILFIVGFILRLVVLARLGDSKMNSDTEAFHILTDPILDPSRICMAFSL   120
Me.TRPMPZQ   SDGWNKLDCTGLILYIVGFTLRLIVLARLGDSKLSENTEAFHILTDPIMDPSRICMAFSL   120
Ht.TRPMPZQ   SDGWNKLDCTGLILYIVGFILRLIVLARLGDSQLSHDTEAFHILTDPIMDPSRICMAFSL   120
             **************:*:** ***:********::. :***********:*********:*

Eg.TRPMPZQ   FVFYIRLMYSFSFHIALGPKLIMIGKMVTNDLIPFMIILTVIMVGYAVAAQSIAYPNGFY   180
Mc.TRPMPZQ   FVFYIRLMYSFSFHIALGPKLIMIGKMVTNDLIPFMIILTVIMVGYAVAAQSIAYPNGFY   180
Em.TRPMPZQ   FVFYIRLMYSFSFHIALGPKLIMIGKMVTNDLIPFMIILTVIMVGYAVAAQSIAYPNGFY   180
Tm.TRPMPZQ   FVFYIRLMYSFSFHIALGPKLIMIGKMVTNDLIPFMIILTVIMVGYAVAAQSIAYPNGFY   180
Ts.TRPMPZQ   FVFYIRLMYSFSFHIALGPKLIMIGKMVTNDLIPFMIILTVIMVGYAVAAQSIAYPNGFY   180
Hm.TRPMPZQ   FVFYIRLMYSFSFHIALGPKLIMIGKMVTNDLIPFMIILAVIMVGYAVAAQSIAYPNGFY   180
Hn.TRPMPZQ   FVFYIRLMYSFSFHIALGPKLIMIGKMVTNDLIPFMIILAVIMVGYAVAAQSIAYPNGFY   180
Me.TRPMPZQ   FVFYIRLMYSFSFHIALGPKLIMIGKMVTNDLVPFMIILTVIMVGYAVAAQSIAYPNGFY   180
Ht.TRPMPZQ   FVFYIRLMYSFSFHIALGPKLIMIGKMVTNDLIPFMILLTVIMVGYAVAAQSIAYPNGFY   180
             ********************************:****:*:********************

Eg.TRPMPZQ   TSEQLSINGTVQGMKFIDTIFSMYTTAYFQMFGDFSLDTLQGEDRNCQNGMCPTKTSRWL   239
Mc.TRPMPZQ   TSEELTINGTVQGMKFIDTIFSMYTTAYFQMFGDFSLDTLQGEDRNCQNGMCPTKTSRWL   239
Em.TRPMPZQ   TSEQLSINGTVQGMKFIDTIFSMYTTAYFQMFGDFSLDTLQGEDRNCQNGMCPTKTSRWL   239
Tm.TRPMPZQ   TSEQLSISKTVQGMKFIDTIFSMYTTAYFQMFGDFSLDTLQGEDRNCQNGMCPTKTSRWL   239
Ts.TRPMPZQ   TSEQLSIDGTVQGMKFIDTIFSMYTTAYFQMFGDFSLDTLQGEDRNCQNGMCPTKTSRWL   239
Hm.TRPMPZQ   TNDKLSINGSVKGMTFIETIFSMYTTSYFQMFGDFSLDTLQGEDRNCENGECPTQTSRWL   239
Hn.TRPMPZQ   TNDKLSINGSVKGMTFIETIFSMYTTSYFQMFGDFSLDTLQGEDRNCENGECPTQTSRWL   239
Me.TRPMPZQ   TNEKLSISETVKGMKFIDTIFSMYTTAYFQMFGDFSLDTLQGEDRNCEYGMCPTKTSRWL   239
Ht.TRPMPZQ   TSEQLSLDGTVQGMKFIDTIFSMYTTAYFQMFGDFSLDTLQGEDRNCQNGMCPTKTSRWL   239
             *.::*::. :*:**.**:********:********************: * ***:*****

Eg.TRPMPZQ   VPIMLGFYVLLTNILMFNLLIAMFSKTYEEIESASTYYWNYQRYQMIADYVRRSPLVPPLII   302
Mc.TRPMPZQ   VPIMLGFYVLLTNILMFNLLIAMFSKTYEEIESASTYYWNYQRYQMIADYVRRSPLVPPLII   302
Em.TRPMPZQ   VPIMLGFYVLLTNILMFNLLIAMFSKTYEEIESASTYYWNYQRYQMIADYVRRSPLVPPLII   302
Tm.TRPMPZQ   VPIMLGFYVLLTNILMFNLLIAMFSKTYEEIESASTYYWNYQRYQMIADYVRRSPLVPPLII   302
Ts.TRPMPZQ   VPIMLGFYVLLTNILMFNLLIAMFSKTYEEIESASTYYWNYQRYQMIADYVRRSPLVPPLII   302
Hm.TRPMPZQ   VPIMLGFYVLLTNILMFNLLIAMFSKTYEEIESASTYYWNYQRYQMIADYVRRSPLVPPLII   302
Hn.TRPMPZQ   VPIMLGFYVLLTNILMFNLLIAMFSKTYEEIESASTYYWNYQRYQMIADYVRRSPLVPPLII   302
Me.TRPMPZQ   VPIMLGFYVLLTNILMFNLLIAMFSKTYEEIESASTYYWNYQRYQMIADYVRRSPLVPPVII   302
Ht.TRPMPZQ   VPIMLGFYVLLTNILMFNLLIAMFSKTYEEIESASTYYWNYQRYQMIADYVRRSPLVPPLII   302
             ***********************************************************:**
```

Species name key:

Eg.TRPM$_{PZQ}$ *Echinococcus granulosus*
Mc.TRPM$_{PZQ}$ *Mesocestoides corti*
Em.TRPM$_{PZQ}$ *Echinococcus multilocularis*
Tm.TRPM$_{PZQ}$ *Taenia multicpes*
Ts.TRPM$_{PZQ}$ *Taenia solium*
Hm.TRPM$_{PZQ}$ *Hymenolepis multiceps*
Hn.TRPM$_{PZQ}$ *Hymenolepis nana*
Me.TRPM$_{PZQ}$ *Moniezia expansa*
Ht.TRPM$_{PZQ}$ *Hydatigena taeniaeformis*

Figure S1. Multiple sequence alignment of the PZQ binding pocket residues in cyclophyllidean cestode TRPM$_{PZQ}$. Species discussed in the manuscript are bolded. The key residues that interact with PZQ are depicted in green text. These key binding residues are identical in all cyclophyllidean cestode TRPM$_{PZQ}$ orthologs examined thus far.

Materials and Methods

General Remarks. No unexpected or unusually high safety hazards were encountered during this work.

Cell Culture and transfection. Activation of the three $TRPM_{PZQ}$ orthologs[1] (*Sm*.$TRPM_{PZQ}$, *Eg*.$TRPM_{PZQ}$ and *Mc*.$TRPM_{PZQ}$) was measured using a Ca^{2+} reporter assay, following transient transfection of codon-optimized constructs (Genscript) into HEK293 cells. The HEK293 cell line (ATCC CRL-1573.3) was authenticated by STR profiling (ATCC), and cells were evaluated for mycoplasma contamination by monthly scheduled testing (LookOut® Mycoplasma PCR Detection Kit, Sigma). HEK293 cells were cultured at 37 ºC in a humidified incubator in Dulbecco's Modified Eagle Medium (DMEM) supplemented with 10% fetal bovine serum (FBS), penicillin (100 units/mL), streptomycin (100 µg/mL), and L-glutamine (290 µg/mL). Cells were transfected using Lipofectamine-2000 at a density of $3x10^6$ cells per petri dish (100 mm diameter) 24 h prior to replating into 384 well plates for functional assays.

Ca^{2+} imaging assays. Ca^{2+} imaging assays were performed using a Fluorescence Imaging Plate Reader ($FLIPR^{TETRA}$, Molecular Devices). HEK293 cells (naïve or transfected) were seeded (20,000 cells/well) in a black-walled clear-bottomed poly-d-lysine coated 384-well plate (Greiner Bio-One) in DMEM growth media supplemented with 10% FBS. After 24 h, growth medium was removed, and cells were loaded with a fluorescent Ca^{2+} indicator (Fluo-4 NW dye, Invitrogen) by incubation (20 µL per well, 1 h at 37°C) in Hanks' balanced salt solution (HBSS) assay buffer containing probenecid (2.5 mM) and HEPES (20 mM). Drug dilutions were prepared in assay buffer, without probenecid and dye, in 'V'-shape 384-well plates (Greiner Bio-one, Germany). After indicator loading, the Ca^{2+} assay was performed at room temperature. Basal fluorescence was monitored for 20 s, then 5 µL of each drug added, and the signal (raw fluorescence units) was monitored over an additional 250 s. Changes in fluorescence were calculated by subtracting the average basal fluorescence (averaged values between 0 to 20 s) from the maximum fluorescence value. These values were normalized to the response of (±)-PZQ at the corresponding $TRPM_{PZQ}$ channel and plotted. Concentration-response curves were generated in GraphPad Prism (*v*. 9.5.1) using the default three-parameter curve fitting and constraining the bottom of the curve to 0%. The reported EC_{50} values represent the mean ± SEM of $n \geq 3$ independent transfections.

Cestode motility assays. Protoscoleces of *E. multilocularis* strain MB17 were isolated from metacestode material grown in experimentally infected gerbils (kindly provided by Prof. Dr. K. Brehm, University of Würzburg, Germany). Extraction and activation of protoscoleces, as well as subsequent motility assays were performed as described previously.[2] In short, protoscoleces were activated by 10% DMSO for 3 h at 37°C, in a humid atmosphere, with 5% CO_2 and left to recover overnight in DMEM (Biochrom, Berlin, Germany) supplemented with 10% FBS (Biochrom) at 37°C, in a humid atmosphere, with 5% CO_2. The next day, 25 protoscoleces were distributed into individual wells of a white, flat-bottom 384 well plate (Huberlab, Aesch, Switzerland). PZQ analogs were added to a final concentration of 100, 30, 10, 1, 0.1, 0.01, and 0.001 µM in 1% DMSO with six replicates each. The assay plate was sealed with a clear view seal foil (Huberlab), and motility was measured in a live cell imaging system (Nikon TE2000E, Hamatsu ORCA ER camera) with the software NIS-Elements AR V4.51 and the JOBS module (Nikon) at 37 °C. Motility of protoscoleces was calculated relative to the DMSO control (1%) at each time point presented and the highest and lowest values were excluded. Results are shown as mean ± SEM. Representative pictures (**Figure 2**) are shown after 12 hours.

Computational Modeling. The validated homology model of (*R*)-PZQ in *Sm*.$TRPM_{PZQ}$, depicting the binding pose in **Figure 3A**, was previously reported[1,3]. **Figure 3B** is a visualization that was generated in the Schrodinger Computational Suite, using the Maestro GUI. The *Sm*.$TRPM_{PZQ}$ homology model was used as the backbone; within this backbone N1388 was mutated to a histidine and T1518 was mutated to a serine to mimic the residues showing variation in *Eg*.$TRPM_{PZQ}$. Within Maestro, the cyclohexane ring of (*R*)-PZQ, from within the *Sm*.$TRPM_{PZQ}$ homology model, was changed to a pyridine ring. These changes resulted in the visualization of **(*R*)-2** adjacent to residues of variation in *Eg*.$TRPM_{PZQ}$. This is a visualization to direct future studies and not an outcome of docking since there is not a validated homology model of *Eg*.$TRPM_{PZQ}$.

[1] Rohr, C. M.; Sprague, D. J.; Park, S. K.; Malcolm, N. J.; Marchant, J. S., Natural variation in the binding pocket of a parasitic flatworm TRPM channel resolves the basis for praziquantel sensitivity. Proc. Natl. Acad. Sci. U. S. A. 2023, 120 (1), e2217732120.
[2] Kovac, J.; Vargas, M.; Keiser, J., In vitro and in vivo activity of R- and S- praziquantel enantiomers and the main human metabolite trans-4-hydroxy-praziquantel against Schistosoma haematobium. Parasites & vectors 2017, 10 (1), 365.s
[3] Park, S. K.; Friedrich, L.; Yahya, N. A.; Rohr, C. M.; Chulkov, E. G.; Maillard, D.; Rippmann, F.; Spangenberg, T.; Marchant, J. S., Mechanism of praziquantel action at a parasitic flatworm ion channel. Sci Transl Med 2021, 13 (625), eabj5832.

Synthetic Chemistry Procedures

General Remarks

All reagents and solvents were commercial grade and purified prior to use if necessary. Solvents were dried by passage through a column of activated alumina as described by Grubbs.[4] DMF was distilled from CaH and stored over activated molecular sieves. Thin layer chromatography (TLC) was performed using glass-backed silica gel (250 μm) plates or glass-backed Biotage® KP-NH plates. UV light, and/or the use of potassium iodoplatinate, potassium permanganate and ninhydrin stains were used to visualize products. MPLC was performed on a Biotage Isolera in conjunction with a Biotage® Dalton 2000 using the conditions indicated. Nuclear magnetic resonance spectra (NMR) were acquired on a Bruker AV-III-500 (500 MHz) spectrometer equipped with a TCI cryoprobe. Chemical shifts were measured relative to residual solvent peaks as an internal standard set to δ 7.26 and δ 77.0 ($CDCl_3$) or δ 2.50 and δ 39.5 (DMSO-d_6). All reported compounds were ≥95% pure by ^{1}H NMR analysis unless otherwise stated. These molecules consistently display broadening and hindered rotation in spectra which is noted for each compound when present. High-resolution mass spectra (HRMS) were recorded at the Indiana University Mass Spectrometry Facility on a Thermo Scientific Orbitrap XL spectrometer by use of the indicated ionization method. A post-acquisition gain correction was applied using reserpine as a lock mass. Compound **8** was previously reported.[5] No unexpected or unusually high safety hazards were encountered during this work.

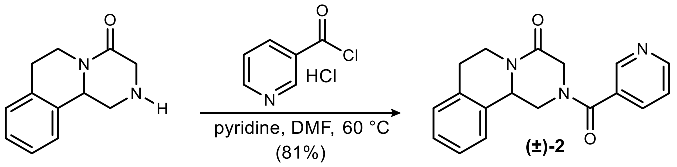

(±)-2-Nicotinoyl-1,2,3,6,7,11b-hexahydro-4*H*-pyrazino[2,1-*a*]isoquinolin-4-one ((±)-2). To a solution of (±)-Praziquanamine (150 mg, 742 μmol) in DMF (2 mL) was added pyridine (5 mL) followed by the acid chloride (198 mg, 1.11 mmol), and the reaction was stirred at 60 °C in a heating mantle for 12 h. After cooling to ambient temperature, the reaction was concentrated *in vacuo*, and the residue was concentrated from toluene (3x) to remove excess pyridine. The residue was dissolved in CH_2Cl_2 and washed with sat aq $NaHCO_3$. The organic layer was passed through a Biotage® phase separator and dry-loaded onto Celite®. MPLC (KP-Amino Duo, 28 g, 20-100% ethyl acetate in hexanes) afforded the product as a colorless solid (185 mg, 81% yield). ^{1}H NMR (500 MHz, DMSO-d_6, 346 K)[6] δ 8.77-8.62 (series of br m, 2H), 7.94 (br d, *J* = 7.8 Hz, 1H), 7.52 (br dd, *J* = 7.7, 5.0 Hz, 1H), 7.30-6.68 (series of br m, 4H), 5.04 (br dd, *J* = 10.1, 3.3 Hz, 1H), 4.63-4.50 (br m, 1H), 4.49-4.14 (br m, 2H), 4.08 (br d, *J* = 17.2 Hz, 1H), 3.45-3.24 (br m, 1H), 2.97-2.76 (series of br m , 3H); ^{13}C NMR (125 MHz, DMSO-d_6, 346 K)[6,7] ppm 167.0, 163.8, 150.9, 147.9, 135.2, 134.9, 133.0, 129.0, 127.1, 126.6, 125.2, 123.5, 54.1, 38.5, 28.2. HRMS (ESI) m/z: $[M+H]^+$ calcd for $C_{18}H_{18}N_3O_2$ 308.1394 found 308.1395.

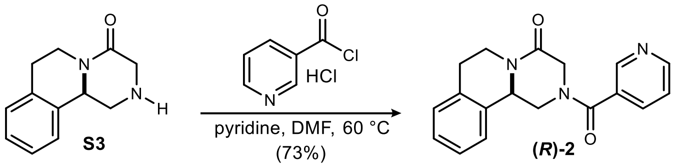

(*R*)-2-Nicotinoyl-1,2,3,6,7,11b-hexahydro-4*H*-pyrazino[2,1-*a*]isoquinolin-4-one ((*R*)-2). To a solution of (*R*)-Praziquanamine (**S3,** >99% ee) (150 mg, 742 μmol) in DMF (2 mL) was added pyridine (5 mL) followed by the acid chloride (198 mg, 1.11 mmol), and the reaction was stirred at 60 °C in a heating mantle for 12 h. After cooling to ambient temperature, the reaction was concentrated *in vacuo*, and the residue was concentrated from toluene (3x) to remove excess pyridine. The residue was dissolved in CH_2Cl_2 and washed with sat aq $NaHCO_3$. The organic layer was passed through a Biotage® phase separator and dry-loaded onto Celite®. MPLC (KP-Amino Duo, 28 g, 20-100% ethyl acetate in hexanes) afforded the product as a colorless solid (167 mg, 73% yield). NMR spectra matched **(±)-2**. HRMS (ESI) m/z: $[M+H]^+$ calcd for $C_{18}H_{18}N_3O_2$ 308.1394 found 308.1395.

---

[4] Pangborn, A. B.; Giardello, M. A.; Grubbs, R. H.; Rosen, R. K.; Timmers, F. J. Safe and Convenient Procedure for Solvent Purification. *Organometallics* **1996**, *15* (5), 1518-1520. DOI: 10.1021/om9503712.
[5] Liu, H.; William, S.; Herdtweck, E.; Botros, S.; Domling, A. MCR synthesis of praziquantel derivatives. *Chem Biol Drug Des* **2012**, *79* (4), 470-477. DOI: 10.1111/j.1747-0285.2011.01288.x.
[6] NMR was run at elevated temperature to sharpen signals which are otherwise too broad to interpret.
[7] Two signals from aliphatic carbons broaden extensively and are not sufficiently visualized in the spectrum.

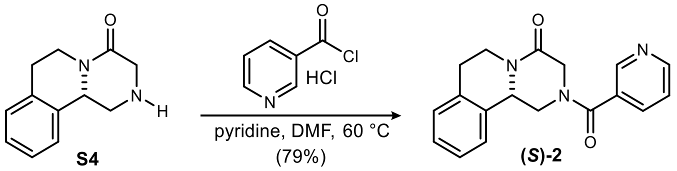

(*S*)-2-Nicotinoyl-1,2,3,6,7,11b-hexahydro-4*H*-pyrazino[2,1-*a*]isoquinolin-4-one ((*S*)-2). To a solution of (*S*)-Praziquanamine (**S4,** 92% ee) (150 mg, 742 µmol) in DMF (2 mL) was added pyridine (5 mL) followed by the acid chloride (198 mg, 1.11 mmol), and the reaction was stirred at 60 °C in a heating mantle for 12 h. After cooling to ambient temperature, the reaction was concentrated *in vacuo*, and the residue was concentrated from toluene (3x) to remove excess pyridine. The residue was dissolved in CH_2Cl_2 and washed with sat aq $NaHCO_3$. The organic layer was passed through a Biotage® phase separator and dry-loaded onto Celite. MPLC (KP-Amino Duo, 28 g, 20-100% ethyl acetate in hexanes) afforded the product as a colorless solid (181 mg, 79% yield). NMR spectra matched **(±)-2**. HRMS (ESI) m/z: $[M+H]^+$ calcd for $C_{18}H_{18}N_3O_2$ 308.1394 found 308.1394.

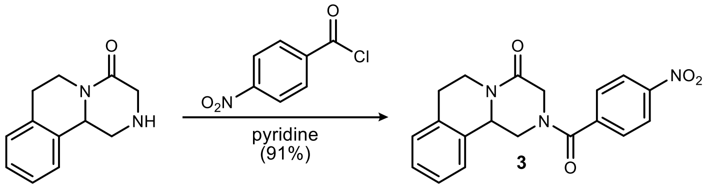

2-(4-Nitrobenzoyl)-1,2,3,6,7,11b-hexahydro-4*H*-pyrazino[2,1-*a*]isoquinolin-4-one (3). To a solution of the amine (100 mg, 494 µmol) in pyridine (5 mL) was added 4-nitrobenozyl chloride (119 mg, 642 µmol), and the reaction was stirred at ambient temperature for 18 h. The reaction was concentrated *in vacuo*, and the residue was dissolved in CH_2Cl_2. The organic layer was washed with 1 M HCl, sat aq $NaHCO_3$, and brine. After passing through a Biotage phase separator, the organic layer was dry-loaded onto Celite®. MPLC (Sfar KP-Amino Duo, 11 g, 12-100% ethyl acetate in hexanes) afforded the amide as a pale-yellow solid (158 mg, 91% yield). R_f = 0.27 (KP-NH, 50% EtOAc/Hexanes); ^{1}H NMR (500 MHz, DMSO-d_6, 346 K)[6] δ 8.30 (d, *J* = 8.6 Hz, 2H), 7.77 (d, *J* = 8.6 Hz, 2H), 7.33-7.00 (br app s, 4H), 5.02 (dd, *J* = 10.3, 3.5 Hz, 1H), 4.54 (br d, *J* = 11.7 Hz, 1H), 4.09-3.96 (br m, 1H), 3.33 (br s, 1H), 3.15 (br s, 1H), 2.96-2.66 (series of m, 4H); ^{13}C NMR (125 MHz, DMSO-d_6, 346 K)[6,7] ppm 167.2, 163.7, 148.4, 141.2, 135.1, 133.0, 129.0, 128.6, 127.1, 126.6, 125.3, 123.8, 54.1, 38.5, 28.2; HRMS (ESI) m/z: $[M+Na]^+$ calcd for $C_{19}H_{17}N_3NaO_4$ 374.1111 found 374.1113.

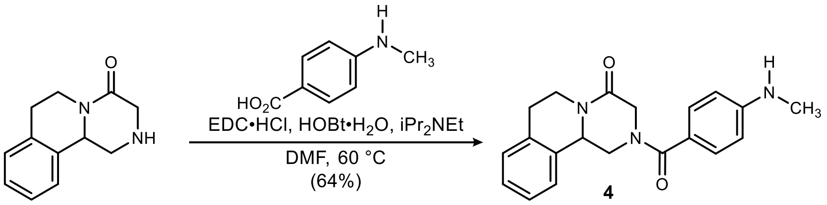

2-(4-(Methylamino)benzoyl)-1,2,3,6,7,11b-hexahydro-4*H*-pyrazino[2,1-*a*]isoquinolin-4-one (4). To a solution of the amine (250 mg, 1.24 mmol) in DMF (4 mL) was added the acid (150mg, 992 µmol), EDC•HCl (190 mg, 992 µmol), HOBt•H_2O (85%, 175 mg, 1.09 mmol), and iPr$_2$NEt (404 µL, 2.32 mmol), and the solution was stirred in a heating mantle at 60 °C for 18 h before concentrating *in vacuo*. The residue was dissolved in CH_2Cl_2 and washed with 1 M HCl, 1 M NaOH, and brine. The organic layer was passed through a Biotage® phase separator and concentrated onto Celite®. MPLC (KP-NH Duo, 28 g, 10-80% ethyl acetate in hexanes) afforded the amide as an amorphous solid in ~90% purity, and the material was used as obtained (213 mg, 64% yield). ^{1}H NMR (500 MHz, $CDCl_3$)[8] δ 7.40 (d, *J* = 8.4 Hz, 2H), 7.34-7.13 (series of m, 4H), 6.62 (d, *J* = 8.4 Hz, 2H), 4.99 (br dd, *J* = 10.5, 2.9 Hz, 2H), 4.82 (br d, *J* = 11.1 Hz, 1H), 4.64 (br d, *J* = 15.7 Hz, 1H), 4.18-3.96 (m, 1H), 3.15-2.84 (series of br m, 3H), 2.89 (s, 3H), 2.83-2.72 (br m, 1H); ^{13}C NMR (125 MHz, $CDCl_3$)[7] ppm 170.8, 164.8, 151.0, 134.9, 132.8, 129.9, 129.3, 127.4, 126.9, 125.4, 122.1, 111.8, 54.9, 38.9, 30.5, 28.8; HRMS (ESI) m/z: $[M+H]^+$ calcd for $C_{20}H_{22}N_3O_2$ 336.1707 found 336.1710.

[8] N-***H*** protons are not observed.

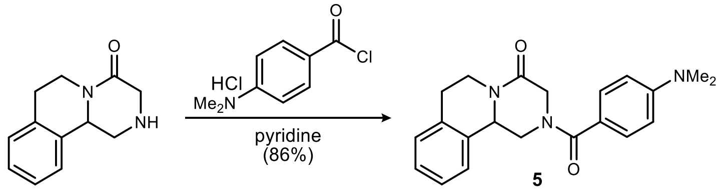

2-(4-(Dimethylamino)benzoyl)-1,2,3,6,7,11b-hexahydro-4*H*-pyrazino[2,1-*a*]isoquinolin-4-one (5). To a solution of the amine (100 mg, 494 µmol) in pyridine (5 mL) was added the acid chloride (136 mg, 741 µmol) and the reaction was stirred at ambient temperature for 18 h and then concentrated *in vacuo*. The residue was dissolved in CH_2Cl_2 and washed with H_2O. The layers were separated, and the organic layer was passed through a Biotage® phase separator and dry-loaded onto Celite®. MPLC (KP-NH Duo, 11 g, 25-100% ethyl acetate in hexanes) afforded the desired amide as a colorless solid (86% yield). ^{1}H NMR (500 MHz, $CDCl_3$) δ 7.45 (d, *J* = 8.8 Hz, 2H), 7.30-7.22 (br m, 3H)[9], 7.19 (d, *J* = 7.0 Hz, 1H), 6.73 (br d, *J* = 8.2 Hz, 2H), 5.00 (br dd, *J* = 10.6, 2.5 Hz, 2H), 4.83 (br d, *J* = 10.8 Hz, 1H), 4.65 (br d, *J* = 16.5 Hz, 1H), 4.11 (d, *J* = 17.8 Hz, 1H), 3.16-2.92 (series of m, 2H), 3.03 (s, 6H), 2.93-2.84 (br m, 1H), 2.82-2.73 (m, 1H); ^{13}C NMR (125 MHz, $CDCl_3$) ppm 170.9, 164.8, 151.8, 134.9, 132.8, 129.7, 129.3, 127.4, 126.9, 125.5, 121.0, 111.5, 54.9, 50.7, 47.6, 40.3, 38.9, 28.8;[10] HRMS (ESI) m/z: $[M+H]^+$ calcd for $C_{21}H_{24}N_3O_2$ 350.1863 found 350.1866.

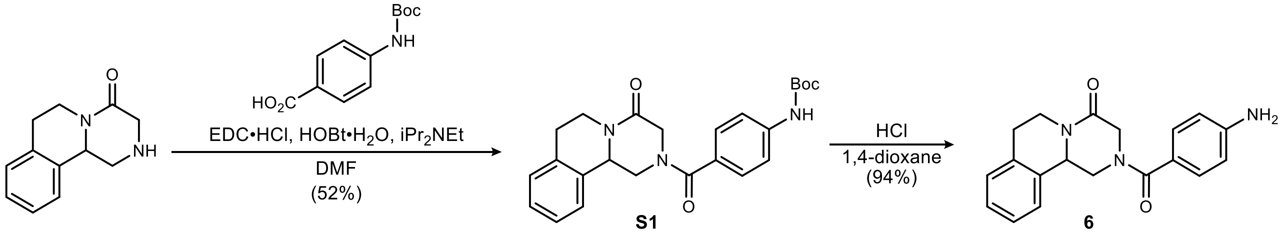

2-(4-Aminobenzoyl)-1,2,3,6,7,11b-hexahydro-4*H*-pyrazino[2,1-*a*]isoquinolin-4-one (6). To a solution of the amine (100 mg, 494 µmol) in DMF (5 mL) was added the acid (176 mg, 741 µmol), EDC•HCl (151 mg, 790 µmol), HOBt•H_2O (85%, 126 mg, 790 µmol) and iPr$_2$NEt (129 µL, 741 µmol). The solution was stirred at ambient temperature for 18 h and then concentrated *in vacuo*. The residue was dissolved in CH_2Cl_2 and washed with 1 M HCl, sat aq $NaHCO_3$, and brine. The organic layer was passed through a Biotage® phase separator and concentrated onto Celite®. MPLC (KP-NH Duo, 28 g, 10-80% ethyl acetate in hexanes) afforded the protected amide (**S1**) as a colorless amorphous solid (108 mg, 52% yield). HRMS (ESI) m/z: $[M+Na]^+$ calcd for $C_{24}H_{27}N_3NaO_4$ 444.1894 found 444.1897.

To a solution of the protected amine (154 mg, 366 µmol) in 1,4-dioxane (2 mL) was added HCl (4 M in 1,4-dioxane, 915 µL, 3.66 mmol), and the reaction was stirred at ambient temperature for 6 h. The reaction was concentrated *in vacuo*, and excess HCl was removed by concentrating from $CHCl_3$ (3x) followed by drying under high vacuum. The residue was dissolved in CH_2Cl_2 and washed with 1 M NaOH. The organic layer was passed through a Biotage® phase separator and concentrated to an amorphous solid that was used without further manipulation (111 mg, 94% yield). ^{1}H NMR (500 MHz, DMSO-d_6, 346 K)[6,8] δ 7.57 (d, *J* = 8.6 Hz, 1H), 7.44 (d, *J* = 8.6 Hz, 1H), 7.27 (d, *J* = 8.4 Hz, 1H), 7.25-7.19 (series of m, 3H), 7.17-7.04 (m, 1H), 6.66 (d, *J* = 8.4 Hz, 1H), 5.00 (br ddd, *J* = 10.2, 10.2, 3.8 Hz, 1H), 4.61 (br dd, *J* = 14.1, 3.8 Hz, 1H), 4.54 (ddd, *J* = 11.7, 4.3, 4.3 Hz, 1H), 4.36 (d, *J* = 17.5 Hz, 1H), 4.02 (br d, *J* = 17.5 Hz, 1H), 3.55-3.45 (br m, 1H), 3.33-3.18 (br m, 1H), 2.97-2.75 (series of m, 2H); ^{13}C NMR (125 MHz, DMSO-d_6, 346 K)[6,11] HRMS (ESI) m/z: $[M+H]^+$ calcd for $C_{19}H_{20}N_3O_2$ 322.1550 found 322.1552.

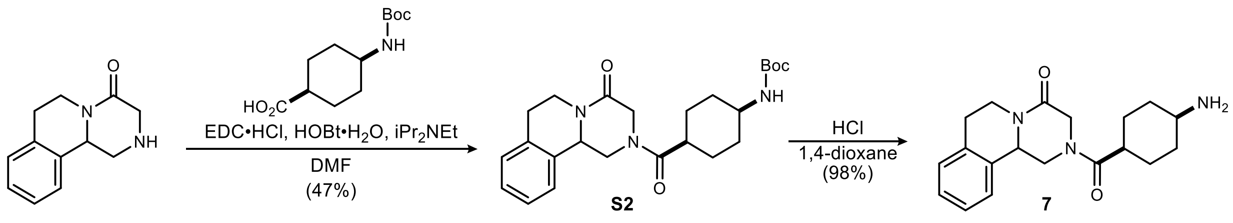

2-(4-Aminocyclohexane-1-carbonyl)-1,2,3,6,7,11b-hexahydro-4*H*-pyrazino[2,1-*a*]isoquinolin-4-one (7). To a solution of the amine (100 mg, 494 µmol) in DMF (5 mL) was added the acid (180 mg, 741 µmol), EDC•HCl (151 mg, 790 µmol), HOBt•H_2O (85%, 126 mg, 790 µmol) and iPr$_2$NEt (129 µL, 741 µmol). The solution was stirred at ambient temperature for 18 h and then concentrated *in*

[9] The signals of three aromatic protons are eclipsed by the residual NMR solvent ($CHCl_3$) at 7.26 ppm.
[10] The signals at 121.0, 50.7 and 47.6 ppm display extreme broadening.
[11] The ^{13}C NMR from this molecule displays extreme hindered rotation or extensive intramolecular hydrogen bonding, even at elevated temperature. As such, the spectrum does not lend itself to an accurate line listing determination, please see image of spectra (Figure S11) instead. In addition to the ^{1}H and ^{13}C NMR spectra, the HRMS image is also attached (Figure S12).

vacuo. The residue was dissolved in CH_2Cl_2 and washed with 1 M HCl, sat aq $NaHCO_3$, and brine. The organic layer was passed through a Biotage® phase separator and dry-loaded onto Celite®. MPLC (KP-NH Duo, 28 g, 10-80% ethyl acetate in hexanes) afforded the amide (**S2**) as a colorless amorphous solid (108 mg, 47% yield). HRMS (ESI) m/z: $[M+Na]^+$ calcd for $C_{24}H_{33}N_3NaO_4$ 450.2363 found 450.2367.

To a solution of the protected amine (140 mg, 328 µmol) in 1,4-dioxane (1 mL) was added HCl (4 M in 1,4-dioxane, 820 µL, 3.28 mmol), and the reaction was stirred at ambient temperature for 6 h. The reaction was concentrated *in vacuo*, and excess HCl was removed by concentrating from $CHCl_3$ (3x) followed by drying under high vacuum. The residue was dissolved in CH_2Cl_2 and washed with 1 M NaOH. The organic layer was passed through a Biotage® phase separator and concentrated to an amorphous solid that was used without further manipulation (105 mg, 98% yield). ^{1}H NMR (500 MHz, DMSO-d_6, 346 K)[6,8,12] δ 7.36 (br s, 1H), 7.30-7.13 (series of m, 3H), 4.88 (br s, 1H), 4.57-4.47 (br m, 1H), 4.42 (br d, J = 17.3 Hz, 1H), 3.56-3.39 (br m, 1H), 3.11-3.00 (m, 1H), 2.97-2.56 (series of m, 5H), 1.94-1.40 (series of m, 9H); ^{13}C NMR (125 MHz, $CDCl_3$)[13] ppm 176.6, 164.6, 135.2, 133.4, 128.9, 127.0, 126.5, 125.5, 72.2, 70.0, 60.4, 54.5, 51.9, 46.3, 38.4, 36.8, 29.8, 28.2, 23.6; HRMS (ESI) m/z: $[M+H]^+$ calcd for $C_{19}H_{26}N_3O_2$ 328.2020 found 328.2022.

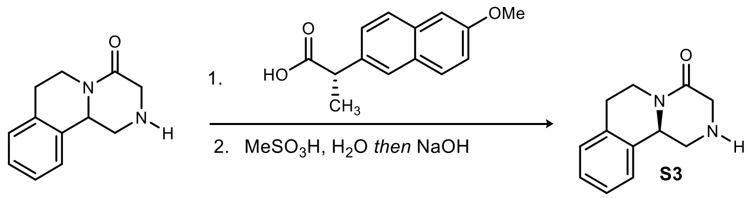

(*R*)-Praziquanamine (S3). (*R*)-Praziquanamine was resolved from the racemate as previously reported[14] employing (*S*)-Naproxen (>99% purity) as the resolving agent. This afforded the enantioenriched material as a colorless solid in >99% ee (Chiralpak AD-H, 20% EtOH/hexanes, 1.0 mL/min, t_r(major) = 15.9 min, t_r(minor) = 18.7 min); ^{1}H NMR spectra matched those previously reported.[14] The amine was acylated without further manipulation.

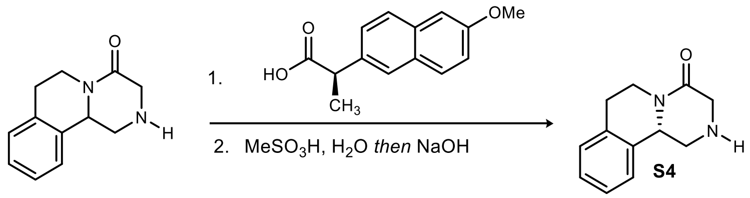

(*S*)-Praziquanamine (S4). (*S*)-Praziquanamine was resolved from the racemate in the same fashion as (*R*)-Praziquanamine (**S3**), employing (*R*)-Naproxen (AKSci, 95% purity) as the resolving agent. This afforded the enantioenriched amine as a colorless solid in 92% ee (Chiralpak AD-H, 20% EtOH/hexanes, 1.0 mL/min, t_r(minor) = 15.9 min, t_r(major) = 18.3 min); ^{1}H NMR spectra matched those previously reported.[14] The amine was acylated without further manipulation.

[12] A small amount of residual chloroform (8.26 ppm) is present in the spectrum after a solvent swap from $CDCl_3$ to DMSO-d_6 (which was made to run elevated-temperature NMR experiments).
[13] A small amount of residual chloroform (79.2 ppm) is present in the spectrum after a solvent swap from $CDCl_3$ to DMSO-d_6 (which was made to run elevated-temperature NMR experiments).
[14] Park, S. K.; Friedrich, L.; Yahya, N. A.; Rohr, C. M.; Chulkov, E. G.; Maillard, D.; Rippmann, F.; Spangenberg, T.; Marchant, J. S. Mechanism of praziquantel action at a parasitic flatworm ion channel. *Sci Transl Med* **2021**, *13* (625), eabj5832. DOI: 10.1126/scitranslmed.abj5832.

Figure S2. ^{1}H NMR (500 MHz, DMSO-d_6) of **(±)-2**

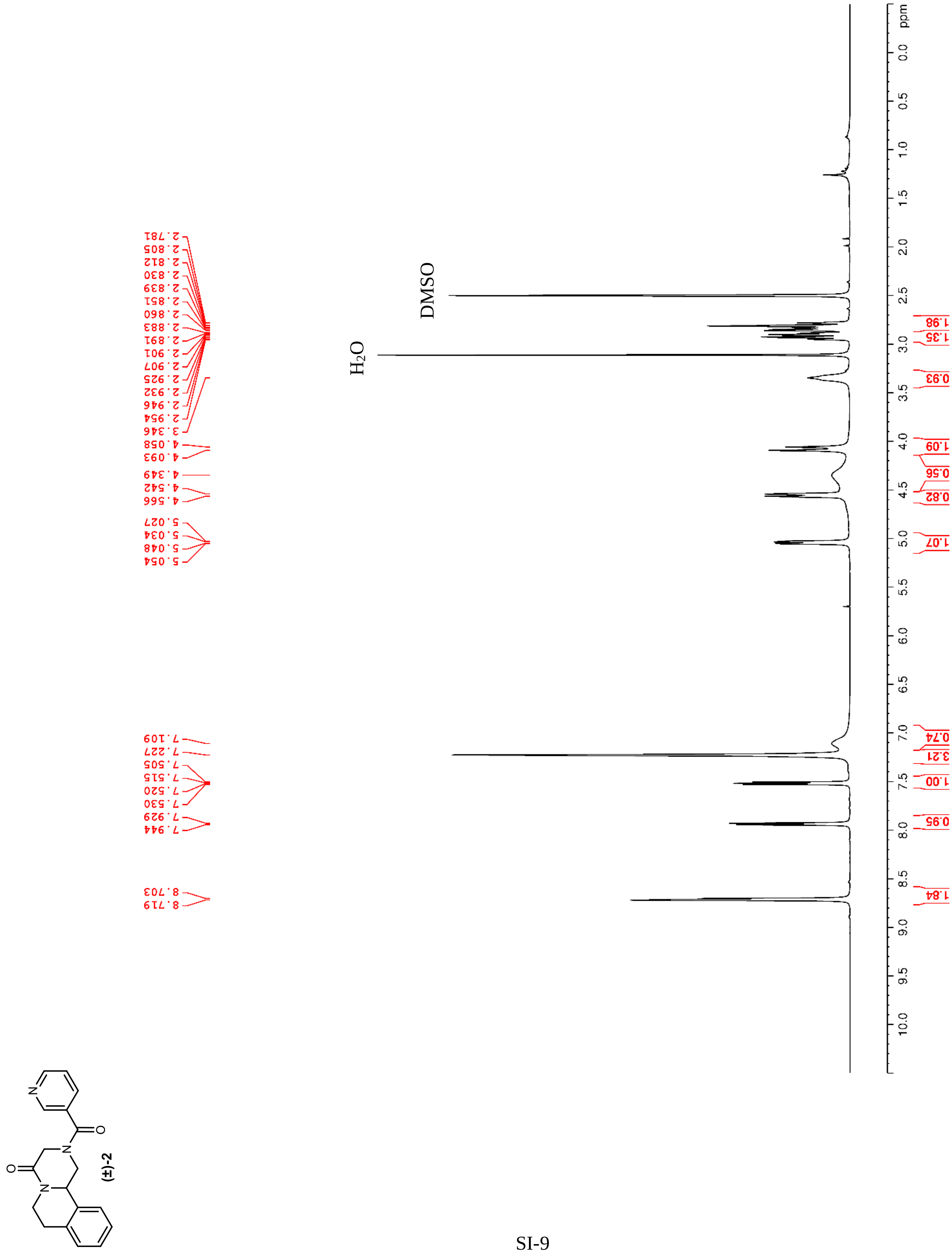

Figure S3. ^{13}C NMR (125 MHz, DMSO-d_6) of **(±)-2**

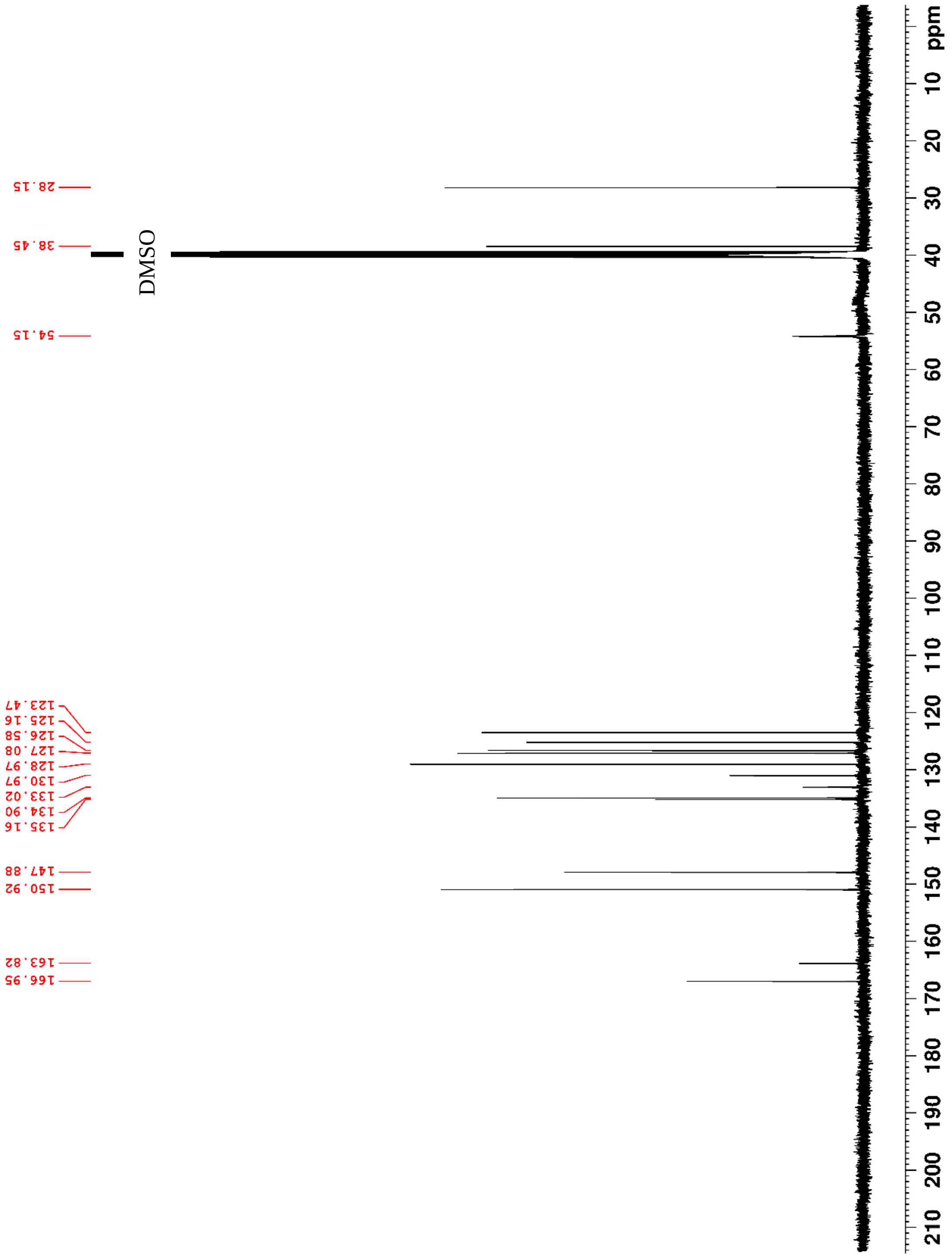

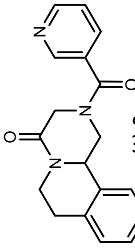

(±)-2

Figure S4. ^{1}H NMR (500 MHz, DMSO-d_6) of **3**

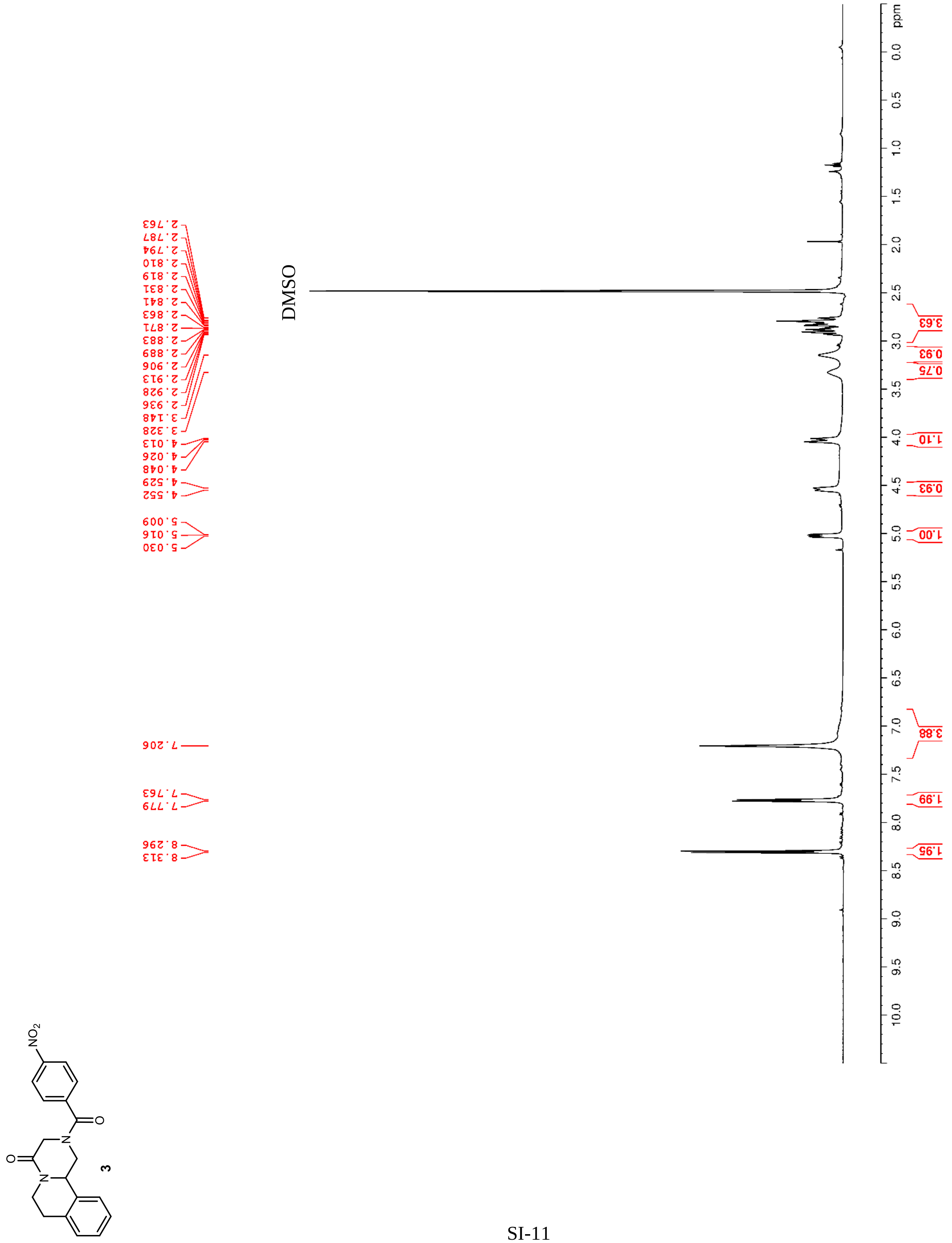

Figure S5. ^{13}C NMR (125 MHz, DMSO-d_6) of **3**

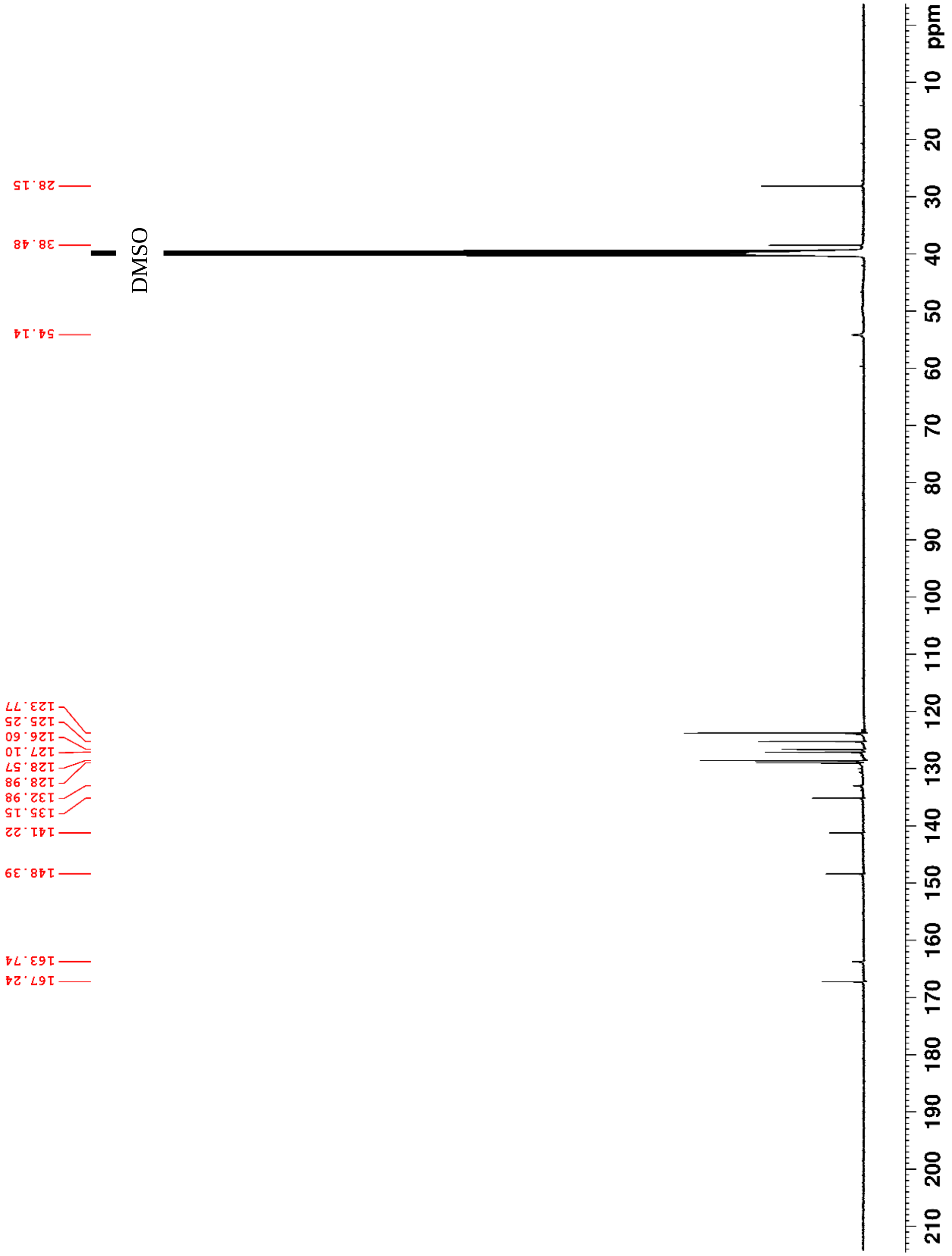

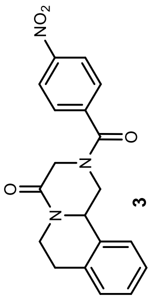

3

Figure S6. ^{1}H NMR (500 MHz, $CDCl_3$) of **4**

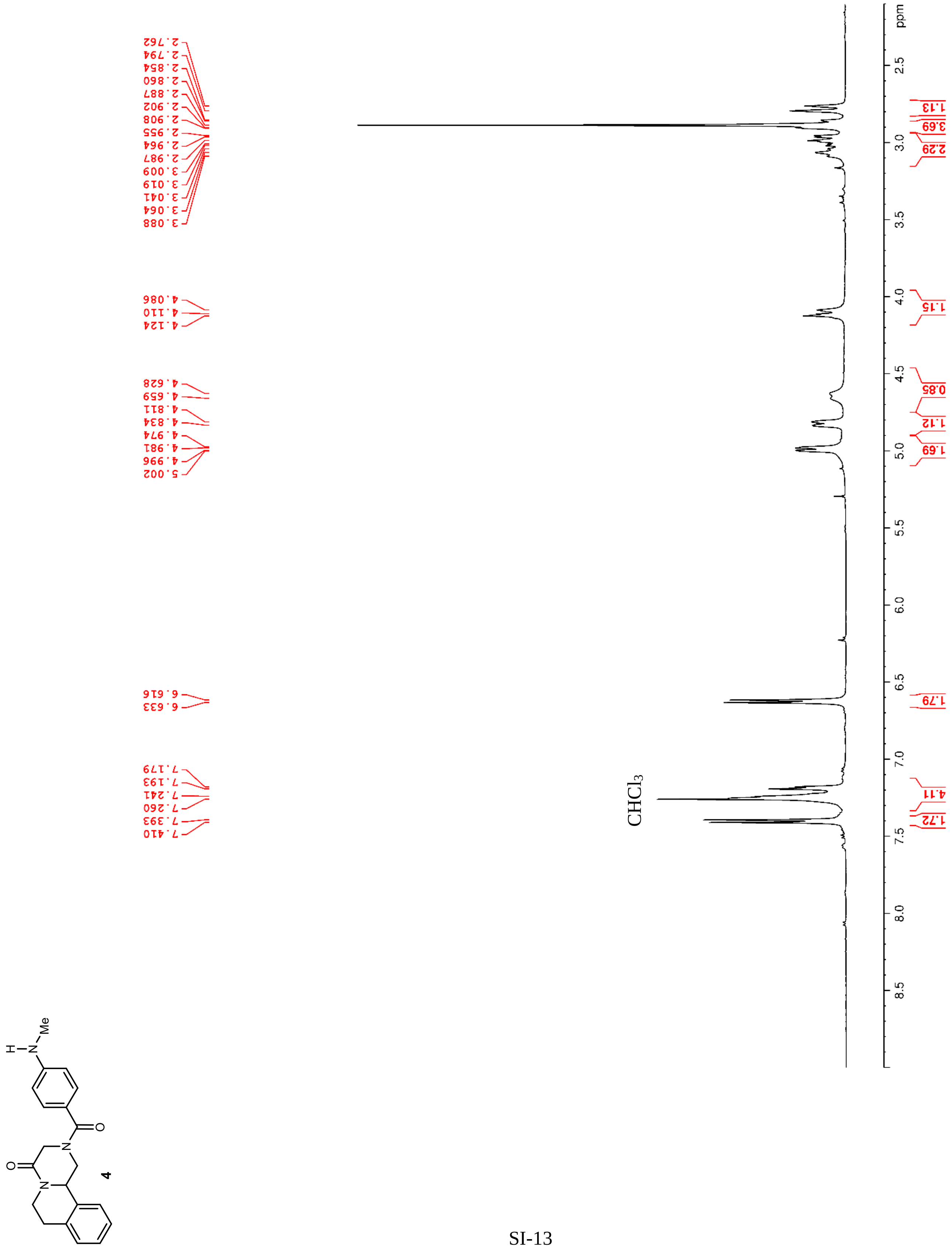

Figure S7. ^{13}C NMR (125 MHz, $CDCl_3$) of **4**

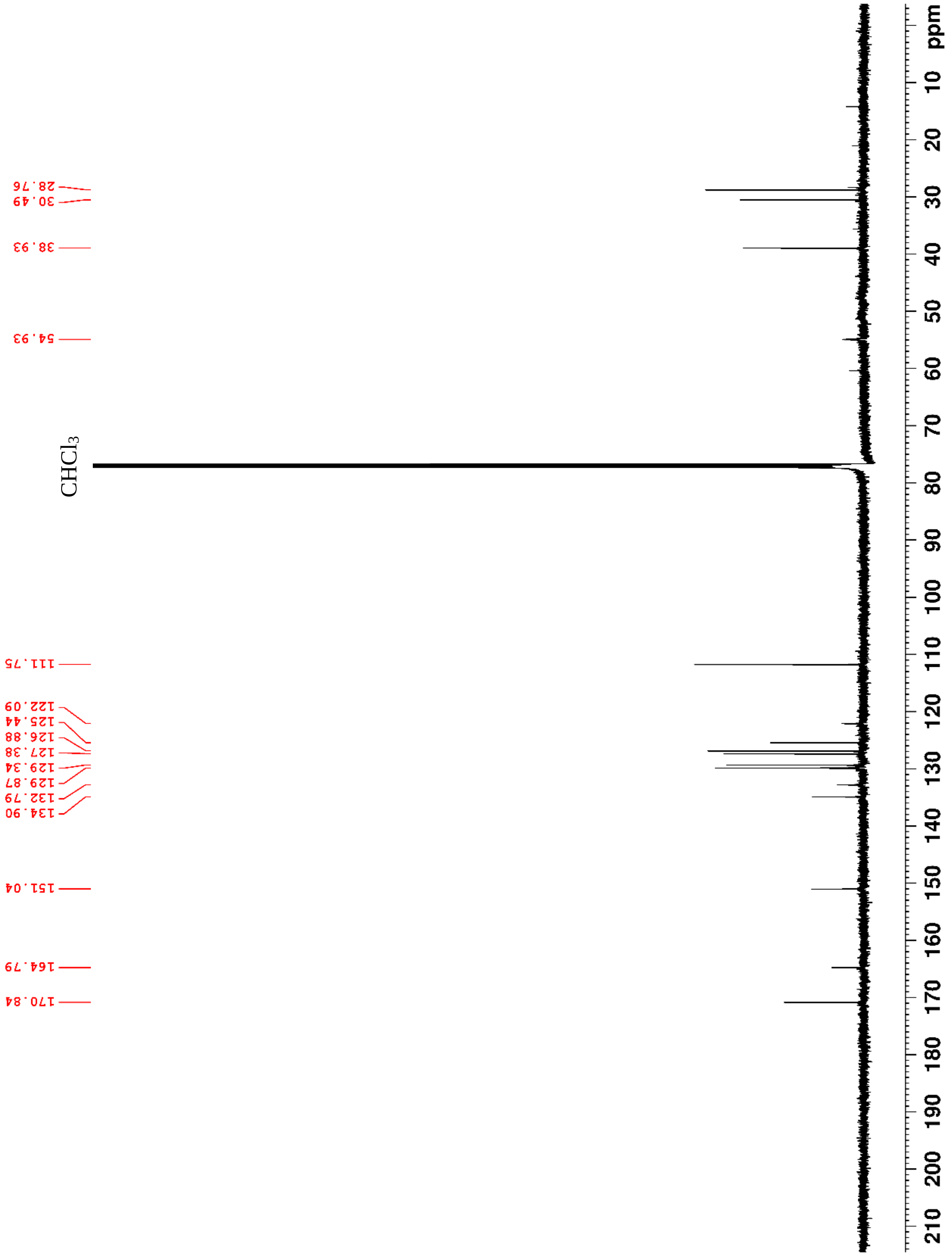

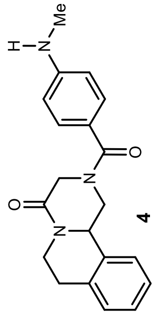

4

Figure S8. ^{1}H NMR (500 MHz, $CDCl_3$) of **5**

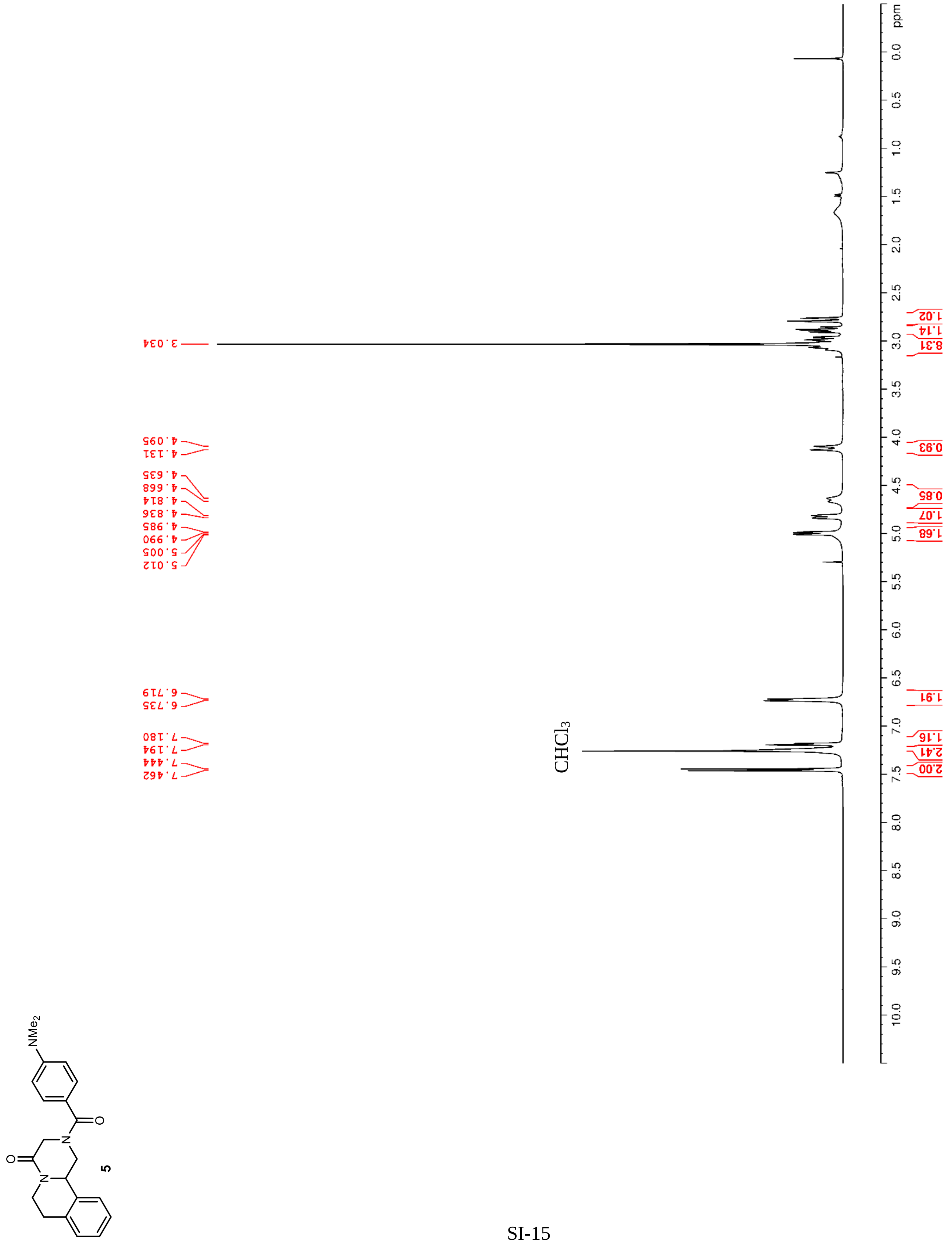

Figure S9. ^{13}C NMR (125 MHz, $CDCl_3$) of **5**

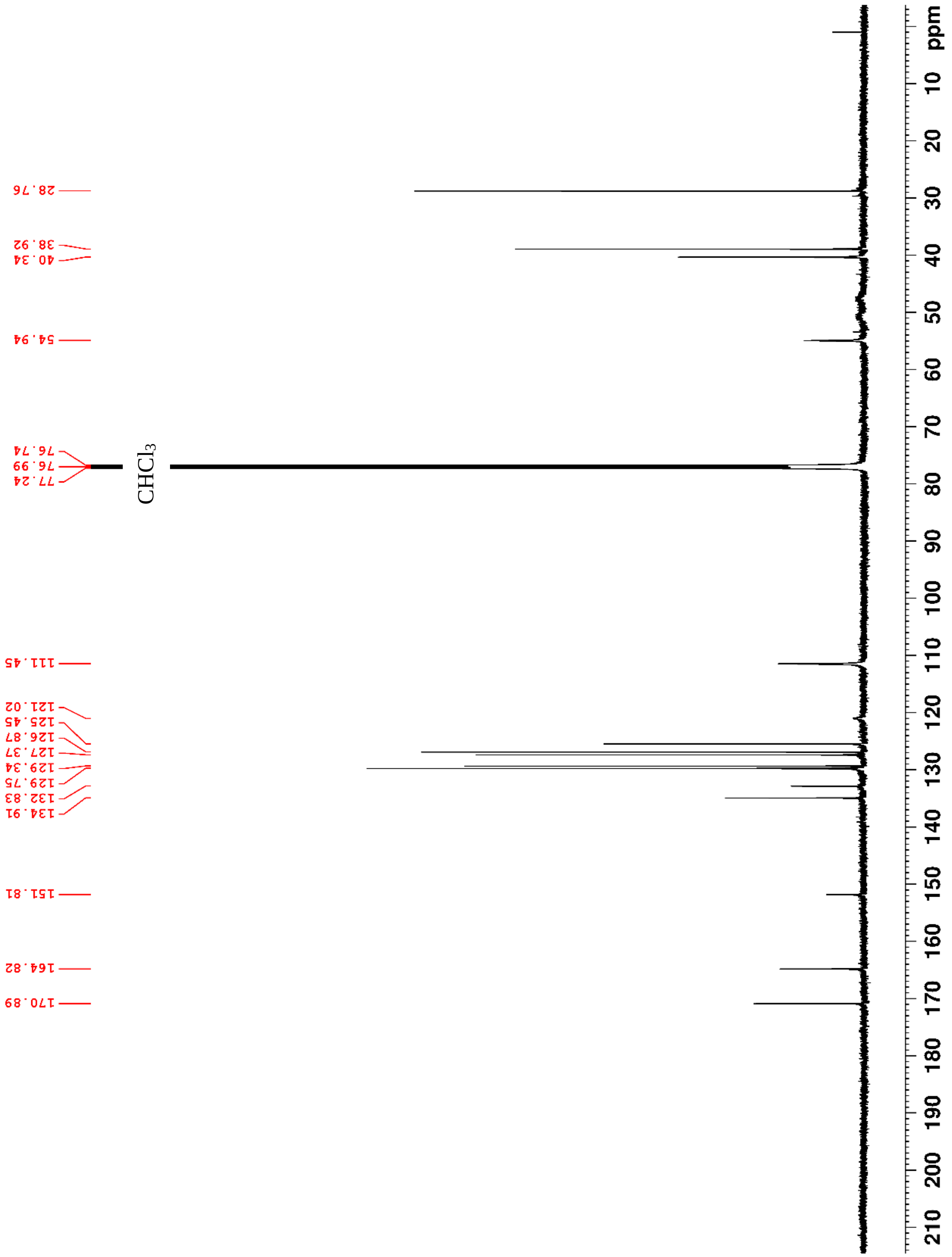

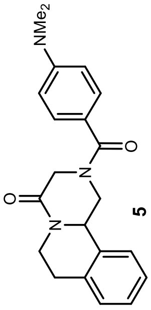

5

Figure S10. ^{1}H NMR (500 MHz, DMSO-d_6) of **6**

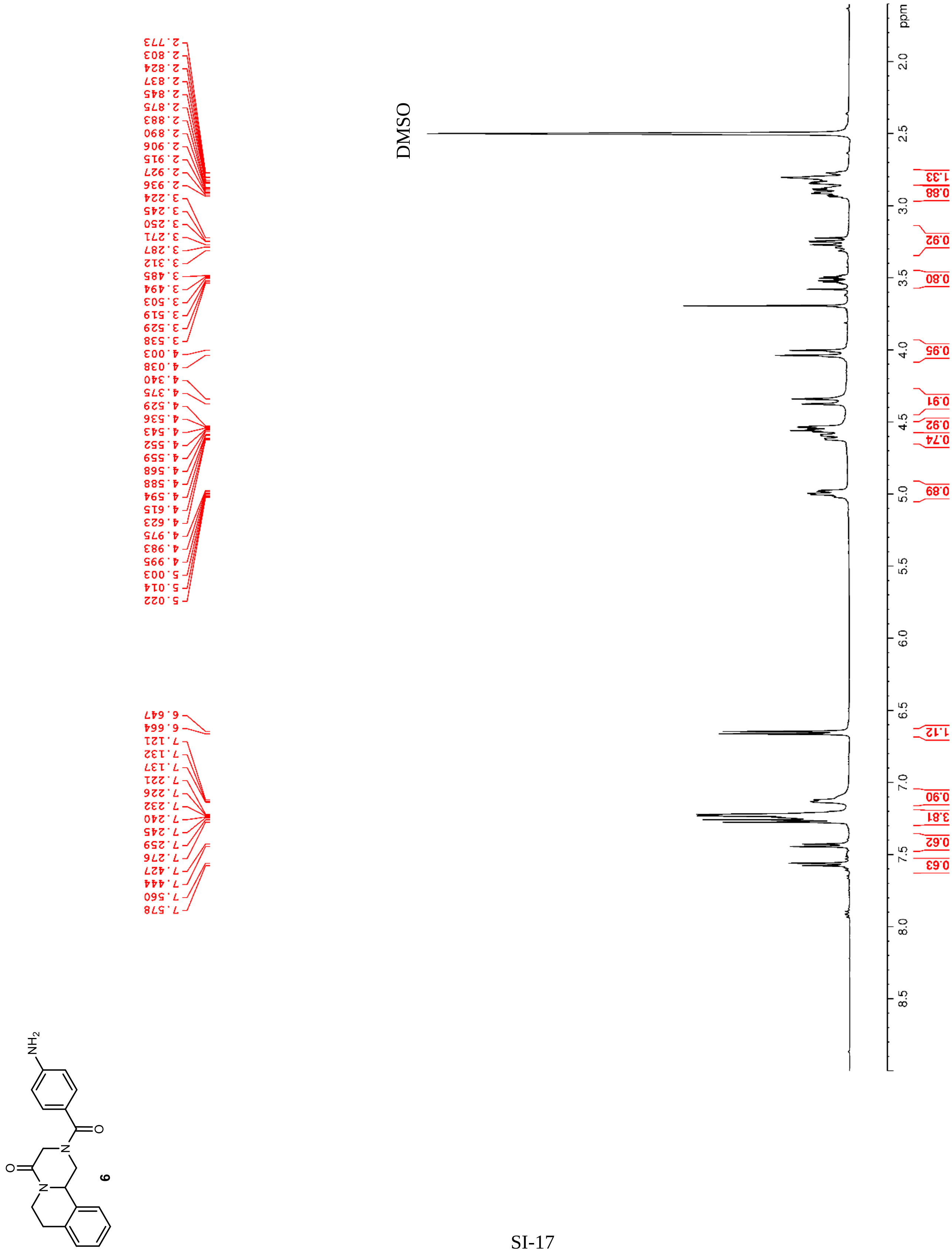

Figure S11. ^{13}C NMR (125 MHz, DMSO-d_6) of **6**

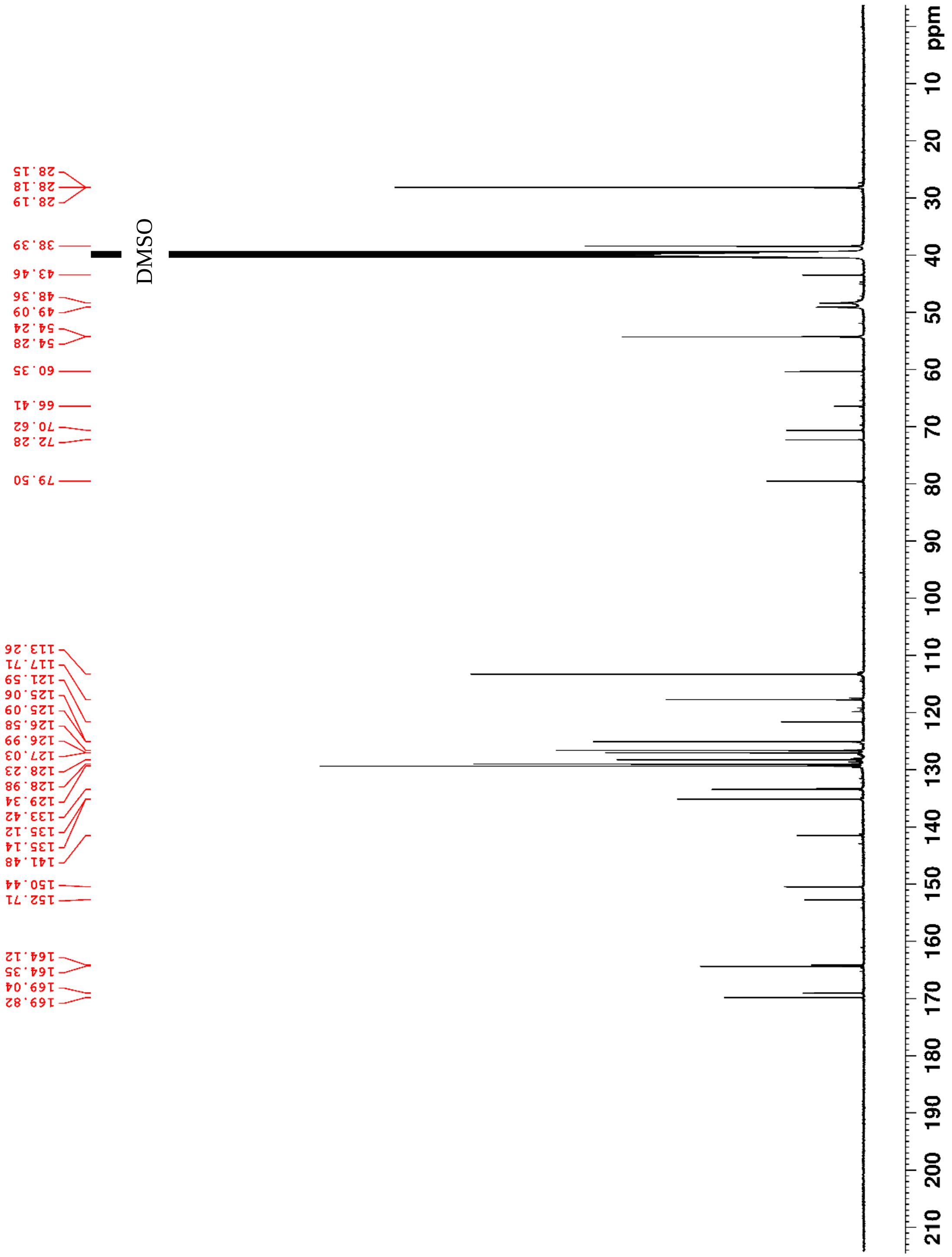

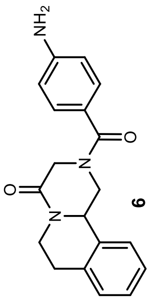

6

Figure S12. HRMS (ESI) of **6**

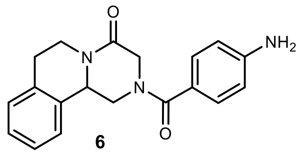

6

Chemical Formula: $C_{19}H_{19}N_3O_2$
Exact Mass: 321.1477

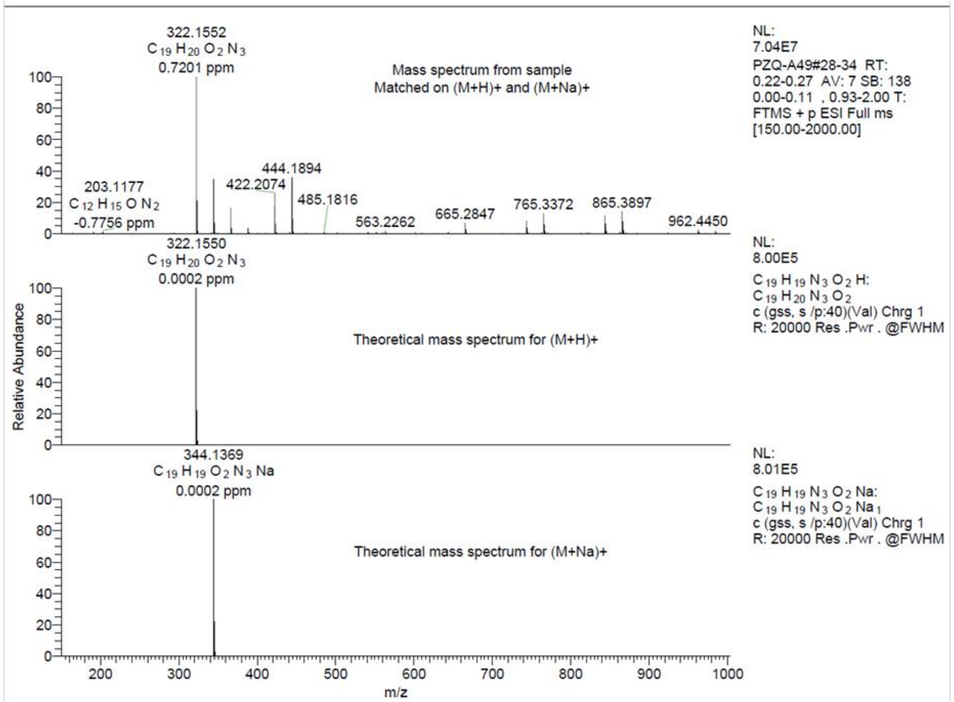

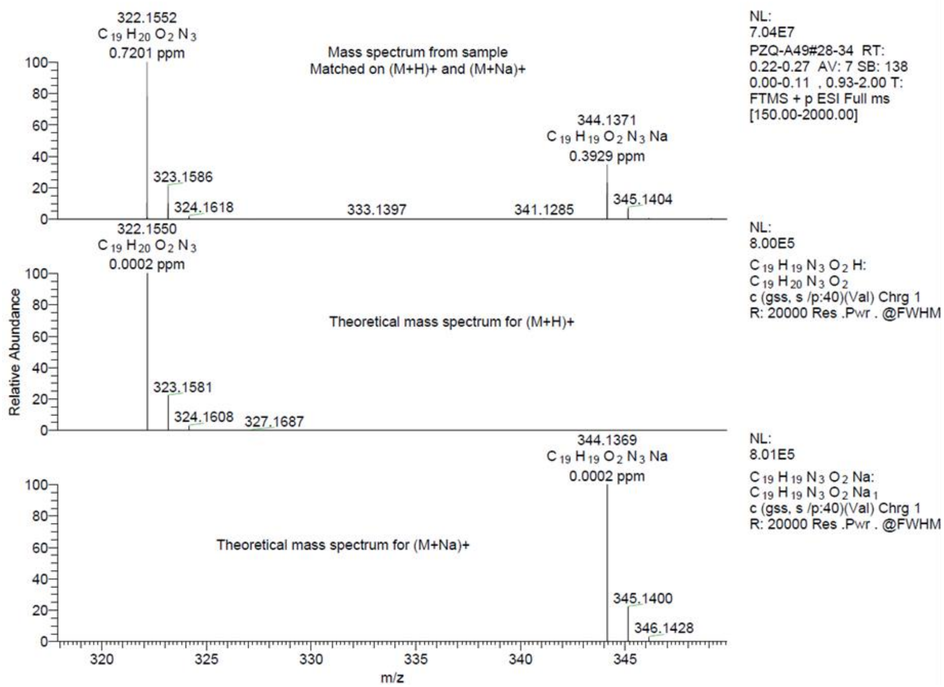

Figure S13. ^{1}H NMR (500 MHz, DMSO-d_6) of **7**

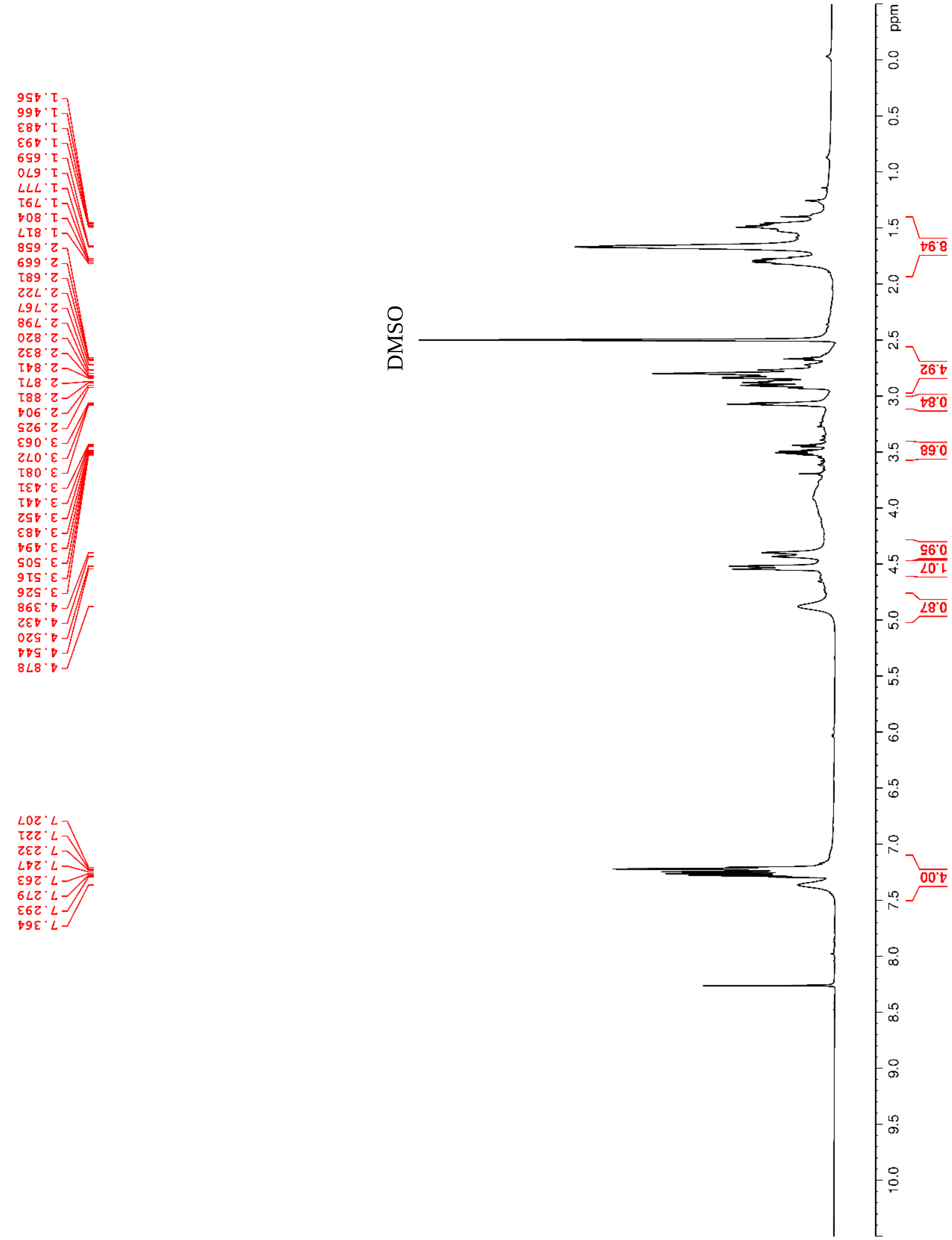

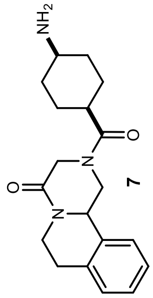

7

Figure S14. ^{13}C NMR (125 MHz, DMSO-d_6) of **7**

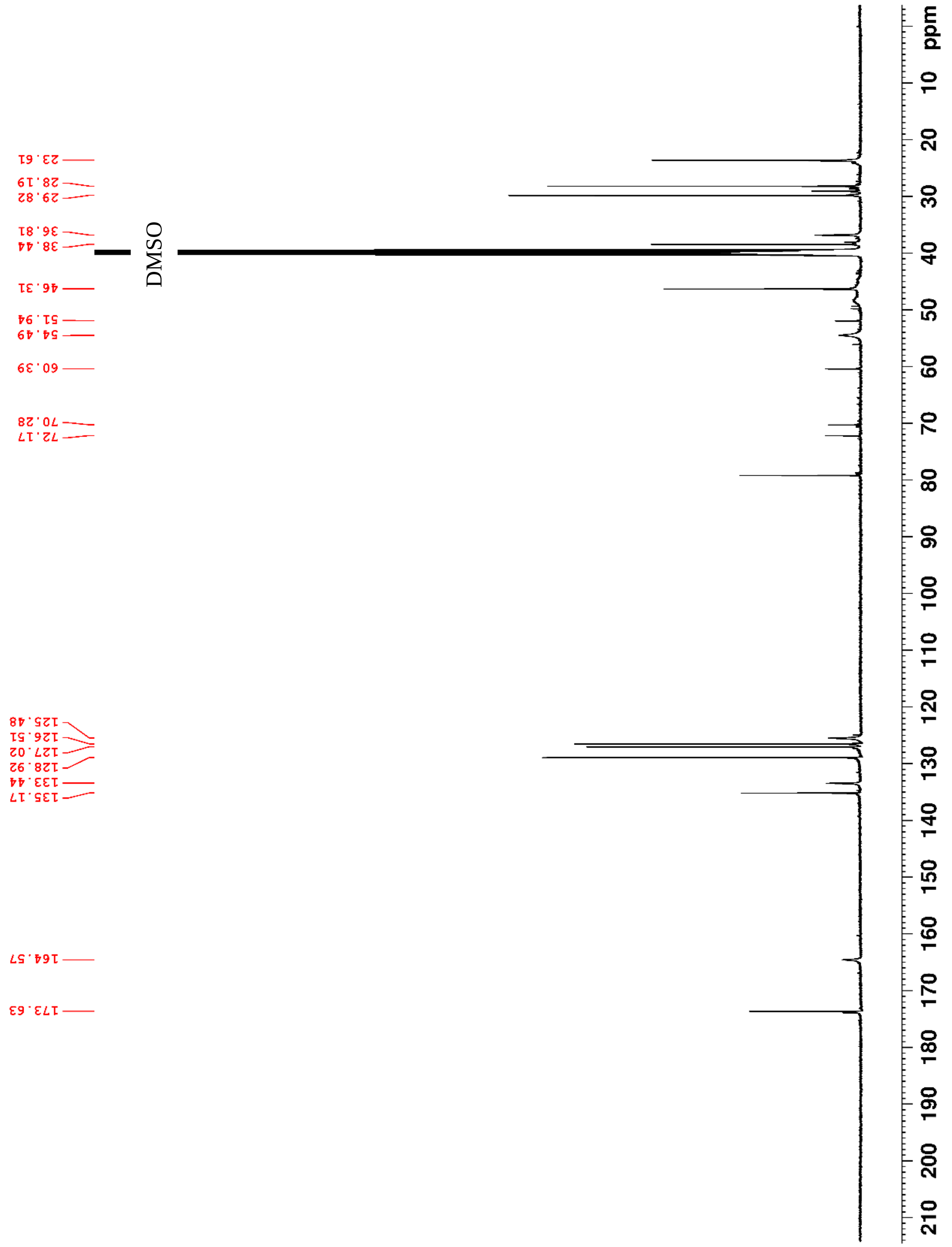

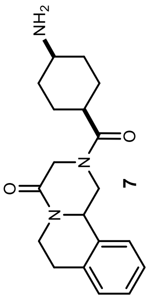

7

Figure S15. HPLC trace of **S3**

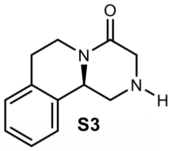

S3

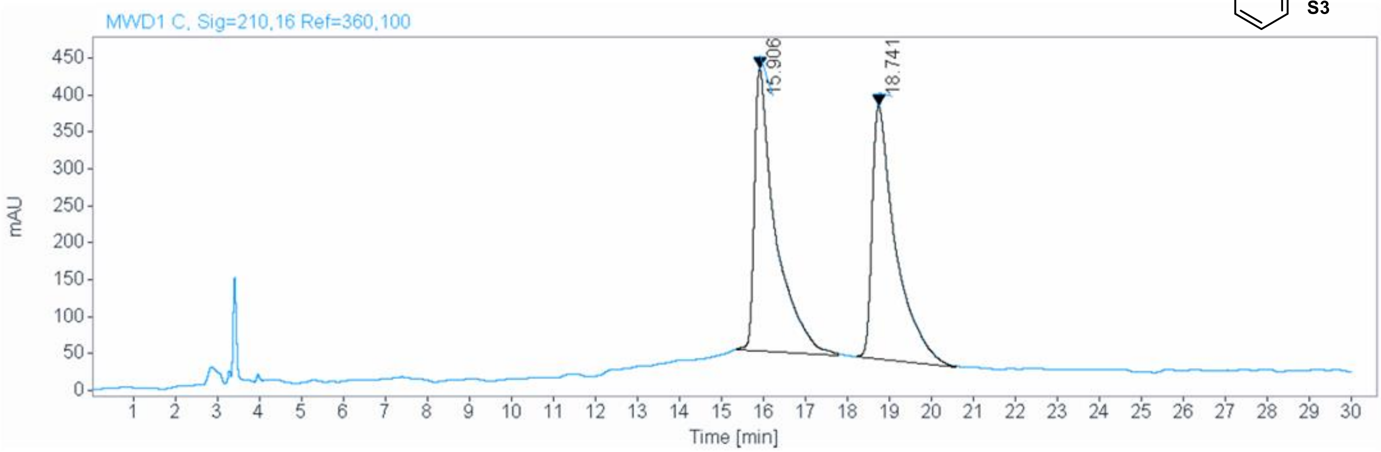

Signal: MWD1 C, Sig=210,16 Ref=360,100

RT [min]	Type	Width [min]	Area	Height	Area%	Name
15.906	MM	0.5798	13307.7188	382.5428	50.1073	
18.741	MM	0.6444	13250.7324	342.7058	49.8927	
		Sum	26558.4512			

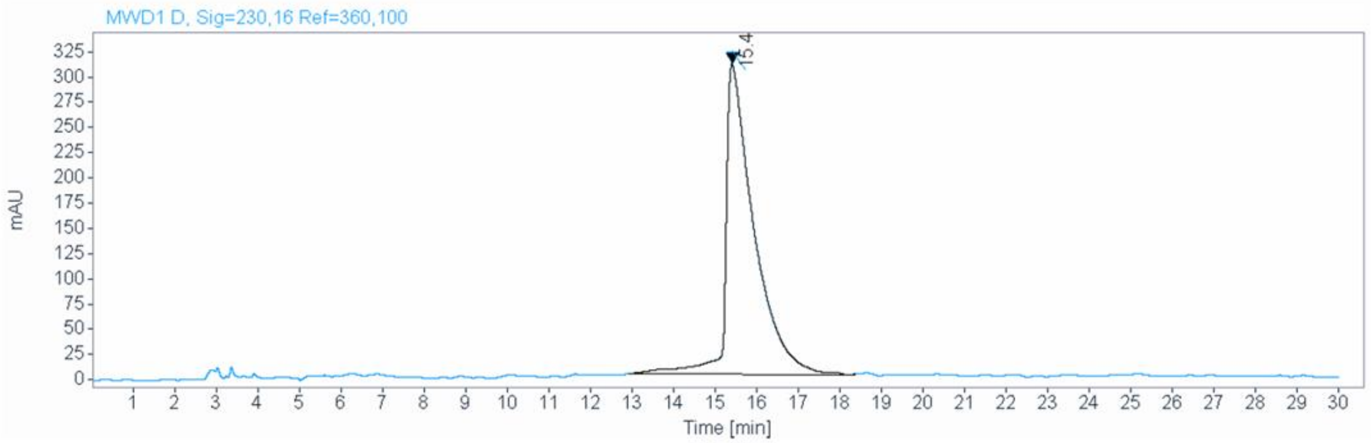

Signal: MWD1 D, Sig=230,16 Ref=360,100

RT [min]	Type	Width [min]	Area	Height	Area%	Name
15.400	MM	0.7840	14462.2813	307.4296	100.0000	
		Sum	14462.2813			

Figure S16. HPLC trace of **S4**

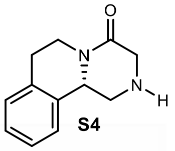

S4

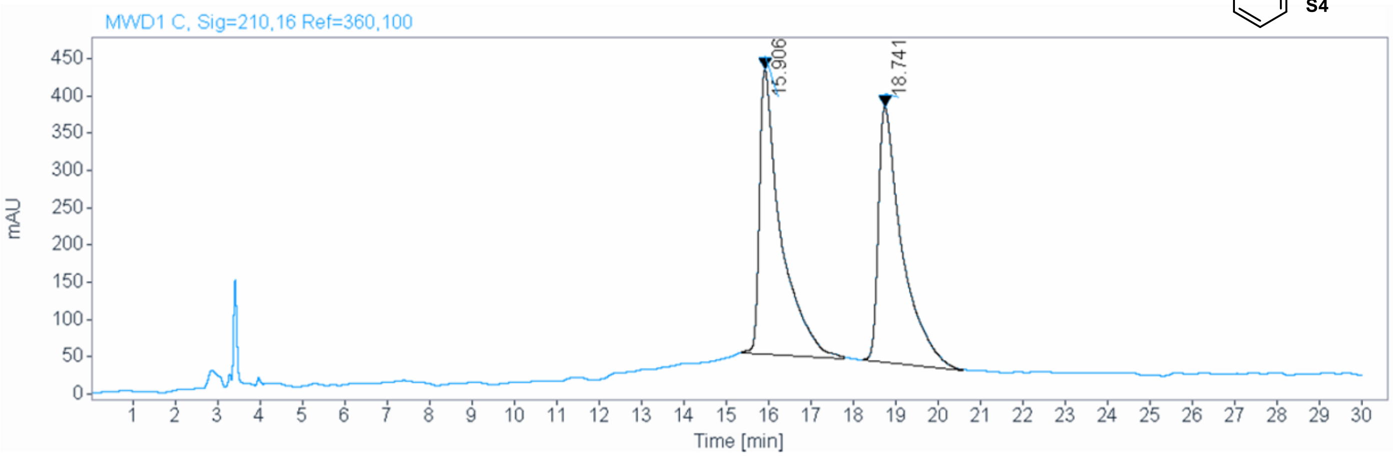

**Signal:**	MWD1 C, Sig=210,16 Ref=360,100					
**RT [min]**	**Type**	**Width [min]**	**Area**	**Height**	**Area%**	**Name**
15.906	MM	0.5798	13307.7188	382.5428	50.1073	
18.741	MM	0.6444	13250.7324	342.7058	49.8927	
		Sum	26558.4512			

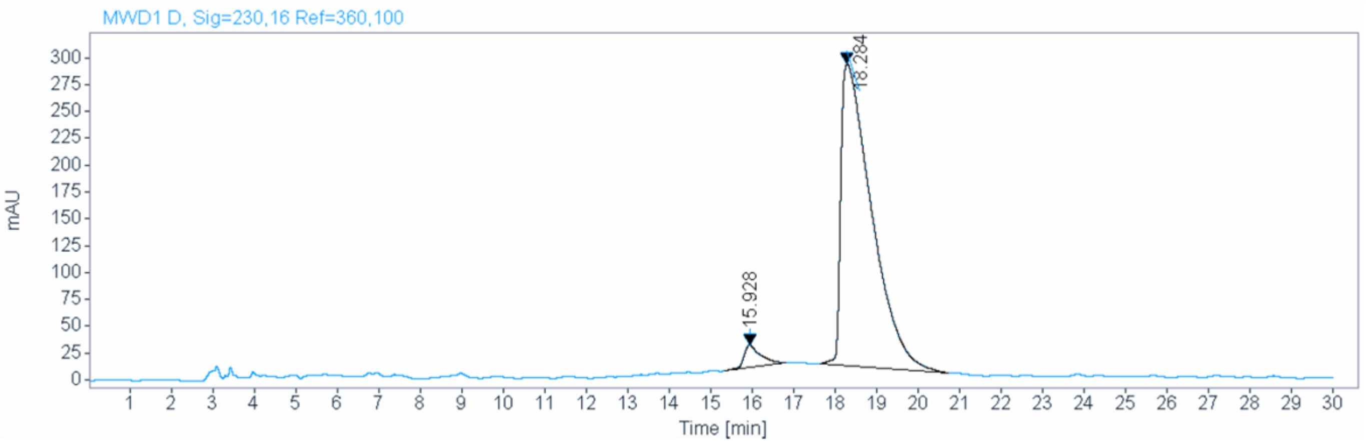

**Signal:**	MWD1 D, Sig=230,16 Ref=360,100					
**RT [min]**	**Type**	**Width [min]**	**Area**	**Height**	**Area%**	**Name**
15.928	MM	0.4604	566.2844	20.4999	3.7677	
18.284	MM	0.8581	14463.6709	280.9143	96.2323	
		Sum	15029.9553			
